# APOBEC3B enhances the efficacy of PARP inhibitors in elimination of ovarian cancer stem cell

**DOI:** 10.1038/s41598-026-35939-y

**Published:** 2026-01-14

**Authors:** Maria Rivera, Lucy Liu, Sabina Enlund, Chae-Eun Lim, Haoran Zhang, Kaifu Yang, Roman Sasik, Leslie A. Crews, Kathleen M. Fisch, Ramez N. Eskander, Frida Holm, Qingfei Jiang

**Affiliations:** 1 Division of Regenerative Medicine, Department of Medicine, University of California, La Jolla, San Diego, CA USA; 2 Moores Cancer Center, La Jolla, San Diego, CA 92037 USA; 3 Sanford Stem Cell Institute, University of California, La Jolla, San Diego, CA USA; 4 Division of Pediatric Oncology and Surgery, Department of Women’s and Children’s Health, Karolinska Institutet, Stockholm, Sweden; 5 Center for Computational Biology & Bioinformatics (CCBB), University of California, La Jolla, San Diego, CA 92093-0681 USA; 6 Department of Obstetrics, Gynecology & Reproductive Sciences, University of California, La Jolla, San Diego, CA USA; 7 Division of Gynecologic Oncology, Department of Obstetrics, Gynecology & Reproductive Sciences, Moores Cancer Center, University of California, La Jolla, San Diego, CA USA; 8 Department of Neuroscience, Karolinska Institutet, Stockholm, Sweden

**Keywords:** DNA damage and repair, DNA replication, Cancer stem cells

## Abstract

Late detection and tumor recurrence are major factors driving the lethality of high-grade serous ovarian carcinoma (HGSOC). PARP inhibitors (PARPi) have achieved significant clinical efficacy by selectively targeting DNA repair deficiencies in HGSOC patients with *BRCA* mutations and homologous recombination deficiency (HRD). However, a subset of patients ultimately develops resistance to PARPi, necessitating alternative effective treatment options. The mutational signatures of APOBEC3 family of DNA deaminases are widespread across a broad array of cancer types. Here, we report that cancer stem cell (CSC)-like tumorspheres exhibit reduced A3B expression compared to non-CSC adherent counterparts. Importantly, inhibition of A3B leads to PARPi resistance, elevated frequency of CSCs, and enhanced expression of stemness factors. In addition, we found that high A3B-expressing cells are under strong replication stress and thus synergize efficiently with PARPi. These studies reveal the important role A3B plays in regulating PARPi response.

**Supplementary Information:**

The online version contains supplementary material available at 10.1038/s41598-026-35939-y.

## Introduction

Poly(ADP-ribose) polymerase (PARP) 1 and 2 play multiple roles in several DNA repair pathways^1^. PARP inhibitors (PARPi) blocks DNA repair and are effective in treating cancer patients with inherited mutations in homologous recombination genes, such as breast and ovarian cancer (e.g*. BRCA1/2* mutated patients)^1,2^. These inhibitors have shown promising efficacy clinically, both as a single agent or following platinum treatment in ovarian cancer patients^3,4^. However, the dramatic increase in clinical PARPi use is inevitably associated with an increase in de novo or acquired resistance to PARPi^5,6^. Patients with PARPi resistance and those who progress while on PARPi maintenance appear to have a poor prognosis and compromised response to subsequent systemic therapy^6,7^. Therefore, overcoming PAPRi resistance would reflect a substantial oncologic milestone in treatment of ovarian cancer.

PARPi were first investigated in malignancies with a high incidence of BRCA1 or BRCA2 mutations, such as breast cancer and ovarian cancer. Ovarian cancer is the most lethal gynecological malignancy^8^. It is estimated that 19,680 new cases of ovarian cancer will be diagnosed in 2024, and ~ 12,740 deaths will occur (seer.cancer.gov). High-grade serous ovarian cancer (HGSOC) is the most common (~ 70%) and most lethal subtype of ovarian cancer^9^. It is frequently diagnosed at stage III or IV, and the 10-year survival rate is less than 30%^10,11^. The poor survival rate is directly related to late-stage diagnosis and relapse following initial surgery and systemic therapy^8,12^. The majority of advanced-stage patients (> 80%) will experience relapse within 5 years^13^. Therefore, understanding the mechanism of progression and therapeutic resistance is essential for reducing mortality and identifying novel drug targets for these patients. It is hypothesized that HGSOC relapses are often driven by cancer stem cells (CSCs) possessing the ability to self-renew and differentiate into bulk tumor tissues upon completion of therapy^14^. Evidence demonstrates that CSCs are responsible for tumor metastasis, disease relapse, and resistance to chemotherapy, suggesting that CSCs are a rational and attractive target for eradicating HGSOC^15,16^.

Whole genomic studies have discovered DNA mutational signatures associated with genetic factors and environmental exposures^17–21^. The dominant enzymatic-driven mutational signature stems from APOBEC3 (Apolipoprotein B mRNA editing enzyme catalytic polypeptide-like 3, A3s)^22^. A3s are a family of DNA deaminases that catalyze the conversion of cytidine to uracil on single-stranded DNA, thereby introducing C-to-T or C-to-G point mutations^23^. As essential components of anti-viral pathways and innate immune responses, A3s restrict the replication of viral particles as well as the retrotransposition of LINE-1 elements in healthy stem cells, thus maintaining the genetic integrity of an organism^24^. Malignant activation of A3s, mainly A3A and A3B, leads to genomic instability and somatic mutations, as evidenced by abundant C-to-T transitions in multiple cancers^18^. Out of the A3 members, A3A and A3B were recently shown to directly initiate or potentiate tumor development in the absence of any driver mutations^25,26^. Of the 30 mutation signatures identified in the cancer genome so far, two are associated with A3 activity and represent the only enzymatic mutation source^27^.

A3B overexpression is frequently observed in both HGSOC cell lines and patient samples^28,29^. These DNA deaminases drive the accumulation of continuous C-to-T mutations that result in cancer evolution and resistance to therapy^30^. Indeed, several drug-resistant mutations were observed in A3 preferred motifs^28,31^ and in driver genes (e.g. *BRCA1* and *TP53*)^32^. In contrast, excess and prolonged activation of A3 enzymes results in DNA mutation overload, eventually driving cells into apoptosis. When expressed at high levels, A3s induce hypermutation of ssDNA, DNA double-stranded breaks (DSB), and cell cycle arrest^33–36^. Moreover, it has been shown that A3B enhances the efficacy of DNA-damaging agents by imposing replication stress^37,38^. However, research reports seeking to understand A3 function in cancer initiation and progression have primarily focused on bulk tumors rather than CSCs. Here, we examine the gene expression patterns of A3s in HGSOC CSC and evaluate the functional roles of A3s in regulating response to PARPi.

## Results

### Differential expression of A3s in HGSOC tumors and immune cells

**Fig. 1:**
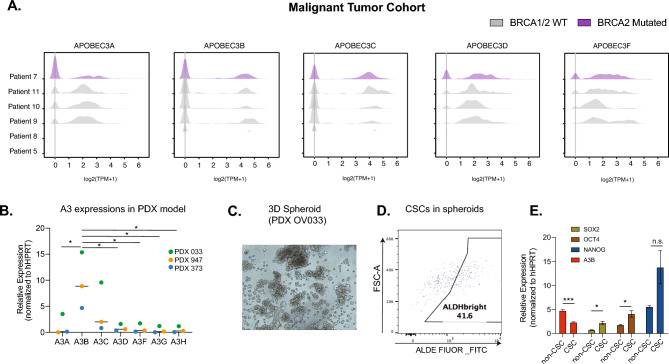
APOBEC3B (A3B) expression is reduced in HGSOC CSCs compared with non-CSCs. (**A**) Ridge plots showing the distribution of APOBEC3s expressions in FACS-enriched tumor cells of 6 HGSOC samples (GSE146026). Patients 5, 8, 9, 10 and 11 are *BRCA1/2* wildtype (colored gray). Patient 7 is BRCA2 mutated and shown in purple. (**B**) Gene expression of APOBEC3s was quantified in 3 PDX models by RT-qPCR. Statistical analysis was performed using one-way ANOVA, followed by Turkey’s multiple comparisons test. *p < 0.05. (**C**) Representative picture of HGSOC 3D spheroids derived from PDX 033 model (20 × magnification). (**D**) Representative flow cytometry showing CSC (ALDH^br^) in PDX 033 spheroid culture. (**E**) Gene expression analysis of A3B in FACS-sorted CSCs (CD133^+^ALDH^br^) and non-CSCs (CD133^-^ALDH^dim^) of PDX 033 3D spheroids (n = 3 experimental triplicates). Error bars represent mean with SEM. *p < 0.05, ***p < 0.001, n.s. not significant, unpaired Student t-test.

To first obtain a broad view of the A3 expression patterns in HGSOC, we used publicly available scRNA-seq datasets^39^ and examined the expression of A3 family members in both tumor cells and immune cell subtypes in HGSOC ascites samples (Figs. 1A and S1A–C). Similar to the originally reported findings^39^, two out of the eight EPCAM^+^CD24^+^ tumor samples were composed of mostly fibroblasts and macrophages rather than malignant tumor cells and were excluded from A3 gene expression analysis (Fig. S1A). The remaining six samples include five samples with wildtype *BRCA1/2* and one with a *BRCA2* mutation (Fig. 1A). In general, A3A is expressed at a lower level compared to A3B, with the exception of sample 8. Surprisingly, A3C is also highly expressed, while A3D and A3F are both expressed at a low level. Both the wildtype and *BRCA2-*mutated tumors express comparable levels of A3B and A3A, although this may be due to the limited number of samples examined. In contrast to tumor cells, immune cells within the ascites rarely express A3A (Fig. S1C). A3B, A3C, and A3G show similar expression patterns in T-cell, B-cell, and dendritic cells, but not in M1 or M2 macrophages.

To confirm the expression patterns of A3s in HGSOC ascites samples, we evaluated the gene expression of A3s in HGSOC patient-derived xenograft (PDX) models using RT-qPCR (Fig. 1B). Among the seven A3 proteins, A3B exhibits the highest level of expression, followed by A3C and A3A, while A3D, A3F, A3G, and A3H are minimally expressed. We also cultured PDX cells as tumorspheres in vitro to enhance CSC properties such as increased expression of stemness genes and aldehyde dehydrogenase activity (ALDH)^40^. PDX 033 was able to readily form tumorspheres with high levels of ALDH^br^ CSC-like population (> 40%, Fig. 1C,D); however, two other PDX samples (PDX 947 and PDX 373) failed to form tumorspheres in the same culture conditions. To compare expression in CSCs and non-CSCs, we sorted out the cells based on ALDH activity (ALDH^br^ or ALDH^dim^) using Fluorescence-activated cell sorting (FACS) (Fig. 1E). As expected, the ALDH^br^ population exhibits a high level of stem cell markers (*OCT4, SOX2, NANOG*), which support self-renewal of CSCs^41^. However, to our surprise, A3B expression was significantly reduced (~ 50%) in ALDH^br^ cells compared to the ALDH^dim^ counterpart, suggesting HGSOC cells downregulate A3B during the transition to a CSC-like state. Together, these data reveal a distinct expression pattern of A3s in HGSOC tumors and immune populations.

### A3B knockdown increases sphere formation ability of HGSOC PDX

We next investigated whether ovarian cancer cell lines suppress A3B expression in sphere culture of SKOV3, TOV-21G, and A2780 cells. The ability to form ALDH^br^ tumorspheres varies among the cell lines, with A2780 spheres containing approximately 67% of ALDH^br^ population, TOV-21G at 16.3%, and SKOV3 at 3.2% of ALDH^br^ cells (Fig. S2A,B). In adherent 2D culture, all cell lines express a higher level of A3B than the other A3 members (Fig. S2C,D). Again, significantly reduced A3B expression was observed in ALDH^br^ tumorspheres compared with adherent cells, while other A3s showed no difference between the adherent and tumorsphere cultures. Together, these data reveal a distinct expression pattern of A3s in HGSOC and suggest HGSOC CSCs maintain a low expression of A3B.

**Fig. 2:**
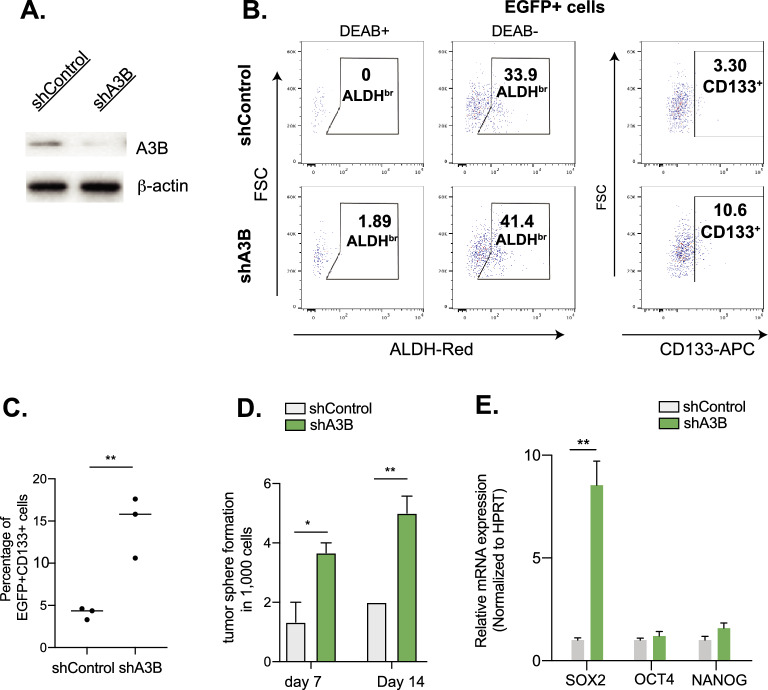
Deletion of A3B reduces CSC frequency. (**A**) Western blot confirming A3B expression following transduction with A3B knockdown lentiviral vector in PDX OV033. (**B**) Representative flow cytometry figures showing ALDH activity and CD133 level in EGFP + OV033 spheroids. (**C**) Quantification of CD133 + populations as shown in (**B**). Experimental triplicates. (**D**) Number of 3D spheroids formed from 1,000 OV033 cells transduced either with lentivirus control (shControl) or A3B knockdown lentivirus (shA3B). Experimental triplicates. (**E**) Relative gene expression of stemness factor *SOX2, OCT4* and *NANOG* in shControl or shA3B cells. Error bars represent mean with SEM. *p < 0.05, **p < 0.01, unpaired Student t-test.

To investigate if A3B plays any functional role in CSCs, we applied shRNA-directed A3B (shA3B) knockdown and confirmed that the shA3B construct does not alter the expression of other A3 members, thereby ruling out any off-target effects (Fig. S2E). We knocked down expression of A3B in PDX 033 and assessed the percentage of the resulting CSC population and its ability to form tumorspheres. PDX 033 was seeded into a 3D sphere culture and transduced with scramble control lentivirus or A3B-shRNA-expressing lentivirus (Fig. 2A). An EGFP marker was applied to isolate successfully transduced cells by flow sorting. To accurately quantify the CSC level, we also included another stemness marker, CD133, alongside the ALDH assay. Cells with A3B knockdown exhibit elevated levels of both CD133 and ALDH^br^ (Fig. 2B,C). In the sphere formation assay, EGFP^+^ shA3B cells were able to form tumorspheres more efficiently than the scramble control (Fig. 2D), indicating that loss of A3B promotes CSC development in HGSOC PDX models.

To gain insights into the molecular mechanism, we examined the expression of self-renewal genes (*SOX2*, *OCT4*, and *NANOG*) in tumorspheres following A3B knockdown (Fig. 2E). These stemness factors are highly expressed in CSCs and often promote tumorsphere formation and CSC self-renewal^41^. In addition, SOX2 is associated with disease relapse and enhanced tumor-initiating potential^41,42^. Interestingly, A3B knockdown significantly increases the expression of *SOX2* (~ tenfold), suggesting loss of A3B reverts CSCs into a more stem-like state.

### A3B promotes response to PAPRi in HGSOC CSCs

**Fig. 3:**
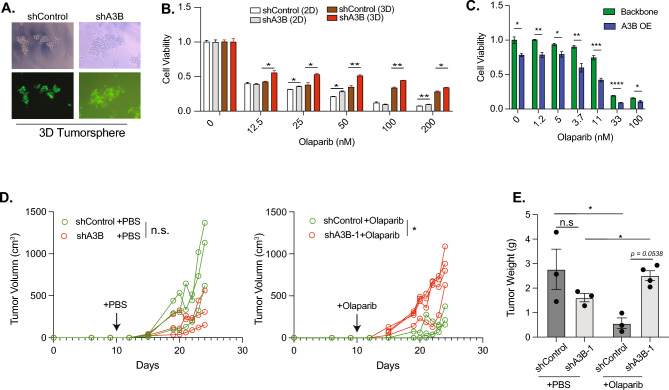
A3B promotes response to PARPi in vitro and in vivo. (**A**) Representative Images of A2780 cells stably transduced with a control lentivirus (shControl) or A3B knockdown lentivirus (shA3B). (** B**) Cell viability was quantified in shControl and shA3B cells cultured in 2D or 3D conditions and treated with Olaparib at various concentrations for 3 days. (**C**) A2780 tumorspheres were transfected with A3B overexpressing plasmid and treated with Olaparib for 3 days. (**D**) Growth curve of A2780 tumor sizes. Xenograft model of A2780 was established by subcutaneous injection on the flank of female Rag2^-/-^γc^-/-^ old female mice (n = 3–4 per group). At day 10 (indicated by the arrow), PBS or Olaparib (50 mg/kg) were intraperitoneally (i.p.) injected daily for 14 days. Each growth curve represents one mouse. n.s. not significant, *p < 0.05, by Two-way ANOVA. (**E**) Tumor weight after 14 days of PBS or Olaparib treatment (n = 3–4 per group). n.s. not significant, *p < 0.05, by Student t-test.

A3-mediated genomic instability is an important source for the generation of drug-resistant clonal mutations during treatment^43^. However, the high mutation loads also come with a cost. A3-expressing cancer cells are constantly under replication stress, which renders them highly susceptible to replication inhibitors such as ataxia telangiectasia and Rad3-related inhibitor (ATRi)^38^. Since PARPi relies on DNA damage to exert efficacy^1,2^, we were interested in examining if A3B plays any role in regulating HGSOC’s response to PARPi. To achieve this goal, we utilized A2780 ovarian cells for A3B knockdown studies, as it has the highest level of A3B expression among the cell lines we tested (Fig. S3A). We generated a stable EGFP^+^ A2780 line with A3B knockdown using the shRNA lentivirus as described above (Figs. 3A and S3B). A2780 cells with A3B knockdown showed a similar proliferation rate compared to the scramble control transduced cells (Fig. S3C). Interestingly, A3B knockdown increases the resistance to the PARPi Olaparib in a dose-dependent manner, and this resistance was more profound in tumorspheres than 2D adherent cells (Fig. 3B). This observation was validated in another ovarian cancer cell line, COV362, which showed a similar resistance upon A3B depletion (Fig. S3D). We further tested if modulating A3B expression would alter sensitivity to ATRi as previously described^38^. Indeed, A3B knockdown reduced sensitivity to ATRi VE-821 in A2780 tumorspheres (Fig. S3E).

Next, we performed overexpression experiments to investigate whether A3B overexpression will sensitize cells to PARPi treatment in three cell lines (Fig. 3C and S3F). Interestingly, A3B overexpression leads to approximately 50% reduction of cell viability in both COV362 and SKOV3 cells (Fig. S3F). In contrast, A2780 cells are able to tolerate A3B overexpression, suggesting that these cells may be more equipped to manage replication stress induced by A3B. A3B overexpression significantly enhanced the efficacy of PARPi in A2780 tumorspheres, thus confirming the results from knockdown studies that A3B activity sensitizes these cells to PARPi treatment (Fig. 3C).

Lastly, we performed in vivo experiments with A2780 cells. A2780 tumorspheres with scramble control or shA3B were transplanted into immunocompromised mice, and Olaparib or PBS was injected at day 10 post-transplant (Fig. 3D,E). In PBS-treated mice, tumor growth was similar between the shControl and shA3B groups, with no significant differences in tumor volume or final tumor weight. This confirmed the in vitro observation that A3B knockdown alone does not affect tumor growth (Fig. S3C). As expected, Olaparib treatment showed a strong anti-tumor effect in control mice compared to PBS control. In contrast, shA3B tumors exhibit accelerated growth and significantly increased final tumor weights compared to the shControl group in the presence of Olaparib. Taken together, our results revealed the importance of A3B in promoting the therapeutic efficacy of PARPi treatment.

### A3B induces replication stress in response to PARPi

**Fig. 4:**
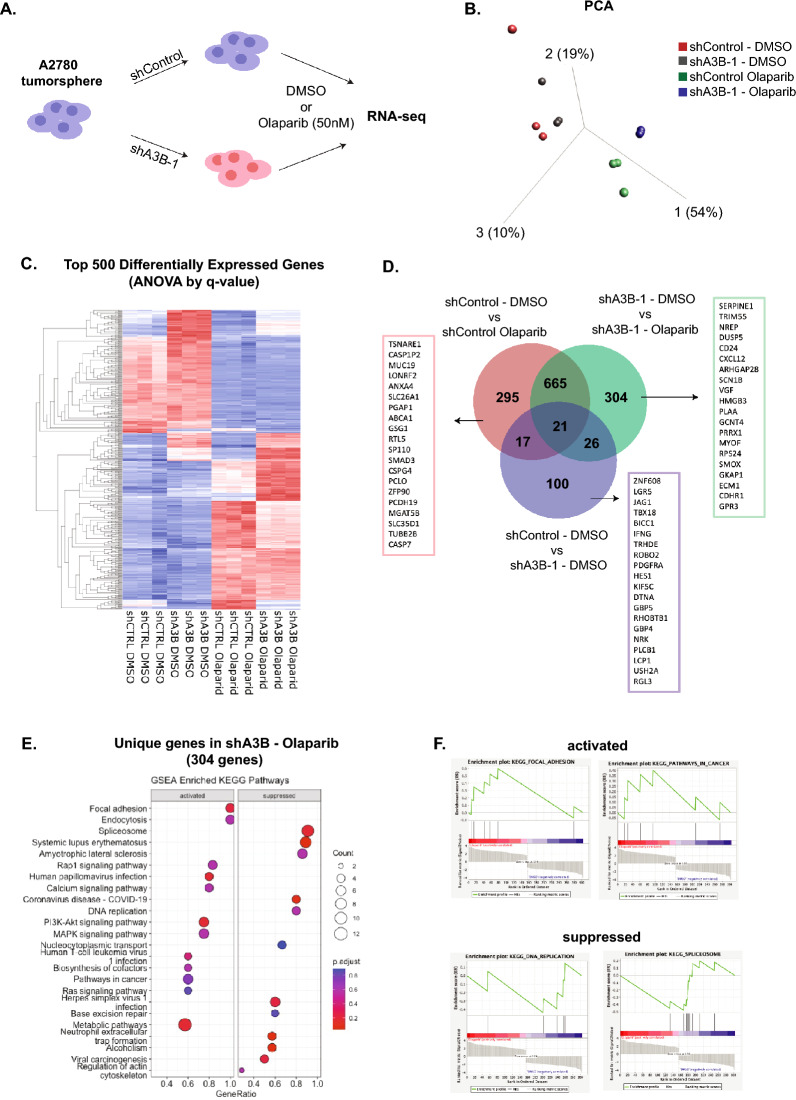
A3B synergistically increase the DNA damages induced by Olaparib in HGSOC tumorspheres. (**A**) Schematic of RNA-seq experimental approach. n = 3 RNA-seq samples per condition. (**B**) PCA plot depicting the grouping of shControl or shA3B-1 transduced tumorspheres treated with DMSO vehicle and Olaparib (50 nM) for 3-days. (**C**) Heatmap of top 500 differentially expressed genes in the four conditions described in A. Significance calculated by ANOVA. (**D**) Venn diagram showing Overlapping and uniquely differentially expressed genes in comparison of Olaparib treatment alone (shControl DMSO vs shControl vs Olaparib), A3B knockdown alone (shControl DMSO vs shA3B-1 vs DMSO), and combination of Olaparib and A3B knockdown (shA3B-1 DMSO vs shA3B-1 vs Olaparib). (**E**) KEGG gene set enrichment analysis of 304 unique genes identified in combination of Olaparib and A3B knockdown. Pathways were ranked based log_2_(fold change) in R using ClusterProfiler. (**F**) KEGG gene set enrichment analysis of 304 unique genes identified in combination of Olaparib and A3B knockdown. Enrichment plots were created in the GSEA application provided by Broad Institute and UC San Diego.

To investigate the exact molecular mechanism underlying A3B function in response to PARPi, we performed RNA-seq analysis of A2780 3D tumorspheres with A3B knockdown in the presence of Olaparib or DMSO vehicle. Tumorspheres were transduced with either scramble control or shA3B-1 knockdown lentivirus, followed by 3 days of treatment with Olaparib or DMSO (Fig. 4A). Principal component analysis (PCA) revealed two distinct clusters of DMSO and Olaparib treatment conditions, which were further separated by A3B knockdown (Fig. 4B). In the DMSO condition, control and A3B knockdown cell clusters are closely grouped, suggesting minimal differences in gene expression. However, the difference is more pronounced between control and shA3B-1 cells under Olaparib treatment. A heatmap of the top 500 differentially expressed genes corroborated the PCA results and showed that the A3B loss alters specific gene expression patterns in combination with Olaparib (Fig. 4C). Further KEGG pathway analysis revealed that the combination of Olaparib and A3B knockdown downregulates several pathways involved in DNA repair regulation, including “mismatch repair” and “base excision repair” compared to Olaparib treatment alone (Fig. S4A,B). In contrast, A3B depletion alone alters only five pathways that are not involved in DNA damage response (Fig. S4C).

We next focused on non-overlapping genes in Olaparib treatment alone, A3B knockdown alone, or a combination of A3B knockdown and Olaparib (Fig. 4D). We identified 295, 100, and 304 differentially expressed genes, respectively. There are also 686 genes shared by the treatment of Olaparib alone and in combination with shA3B, which accounts for the majority of the 998 differentially regulated genes in Olaparib alone. In contrast, far fewer overlapping genes were identified in tumorspheres with A3B loss alone under Olaparib treatment (64 out of 164), suggesting that A3B does not contribute significantly to HGSOC CSC activity in the absence of DNA damage. The KEGG pathway analysis revealed that the unique targets in combination A3B knockdown and Olaparib treatment showed suppression of the spliceosome, DNA replication, base excision repair, and several inflammation-related pathways (for example, systemic lupus erythematosus and COVID-19) (Fig. 4E,F). Together, these data suggest that A3B elevates DNA replication stress to synergize with PARPi.

### A3B knockdown reduces Olaparib-induced DNA damage response

**Fig. 5:**
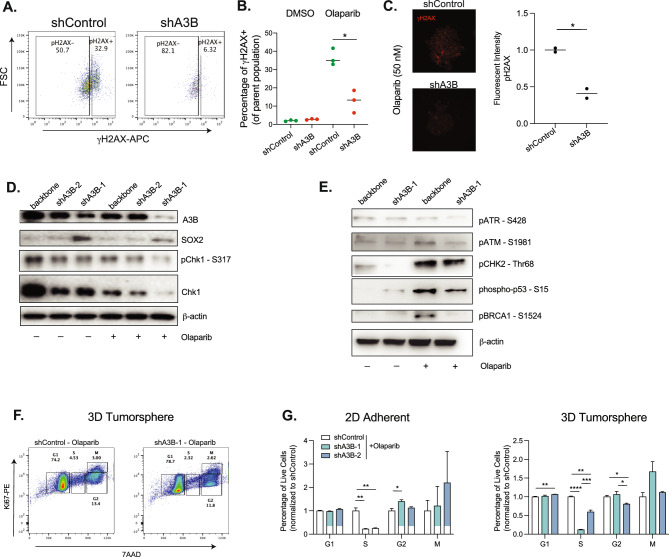
A3B loss reduces excess DNA damage induced by Olaparib. (**A**, **B**) Representative flow cytometry (**D**) and quantification (**E**) of percentages of γH2AX^+^ cells in shControl or shA3B cells after Olaparib treatment (200nM). (**C**) Representative immunofluorescent imaging and quantification of γH2AX signals in shControl and shA3B tumorspheres after treatment with Olaparib (50 nM). (**D**) Western blot analysis of A3B, SOX2, Chk1 and p-Chk1 in A2780 tumorspheres with A3B knockdown followed by DMSO or Olaparib treatment (100 nM) for 3 days. β-actin was used as loading control. (**E**) Western blot analysis of pATR, pATM, pCHK2, p-p53, and pBRCA1 in control or shA3B-1 knockdown cells treated with Olaparib (100 nM) for 3 days. (**F**) Representative cell cycle analysis of 7AAD and Ki67 in A2780 3D sphere culture. (**G**) Cell cycle analysis of A2780 2D adherent cells or tumorspheres treated with DMSO control or Olaparib (50 nM) for 3 days. N = 3 experiments. *p < 0.05, **p < 0.01, ***p < 0.001, and ****p < 0.0001 unpaired Student t-test.

We next examined the level of a double-strand DNA break marker γH2AX (ser139) in Olaparib-treated tumorspheres by flow cytometry. Indeed, shA3B CSCs have less γH2AX compared to backbone-transduced cells (~ 50% reduction) (Figs. 5A and 5B). To directly visualize the double-strand breaks, immunofluorescent analyses for γH2AX were performed on both 2D adherent cells and 3D tumorspheres treated with a low dose of Olaparib (50 nM). Cells with A3B knockdown had lower levels of γH2AX loci than control cells, regardless of 2D or 3D culture conditions, suggesting A3B synergizes with the cytotoxicity of Olaparib via enhanced DNA damage in both CSCs and non-CSCs (Figs. 5C and S5A,B).

To evaluate the changes associated with PARP pathways in response to A3B knockdown, we examined the expression of PARP signaling (PARP1 and cleaved PARP1) and stemness factor SOX2 in the presence and absence of Olaparib (Figs. 5D,E and S5C,D). We also developed an additional shRNA lentivirus to knockdown A3B (shA3B-2) and renamed the current shRNA as shA3B-1. Both constructs efficiently knock down A3B, and shA3B-1 proves more effective in depleting A3B in 3D tumorspheres (Fig. 5D). Olaparib treatment alone results in reduced A3B expression, likely due to the selective depletion of A3B-high expressing cells (Fig. 5D). Consistent with the increased SOX2 expression in PDX 033 (Fig. 2E), we observed elevated SOX2 levels in A3B-knockdown A2780 cells (Fig. 5D). In addition, A3B knockdown consistently reduced the level of cleaved PARP and γH2AX compared to Olaparib-treated control cells, and this effect is more pronounced in shA3B-1 transduced cells than shA3B-2 conditions (Fig. S5C,D).

We also examined the DNA damage response genes in the presence of A3B knockdown and Olaparib treatment. Interestingly, A3B inhibition led to reduced levels of CHK1 S317 phosphorylation in DMSO control-treated cells, which is normally associated with S/G_2_ cell cycle checkpoint^44^ (Fig. 5D). Total CHK1 was detected in all conditions, but was also decreased in the A3B knockdown cells compared with control counterparts. In response to PARPi treatment, CHK1 S317 levels are further reduced, suggesting that loss of A3B reduces replication stress in S/G_2_ cell cycle stages. Since A3B inhibition reduced Olaparib-induced dsRNA break, we also examined the ATM signaling pathway (Fig. 5E). dsDNA breaks activate ATM, which triggers phosphorylation of downstream effector proteins, including checkpoint kinase CHK2, tumor suppressor p53, and BRCA1^45,46^. The resulting cell cycle arrest can occur in both G_1_/S and G_2_/M phases. A3B knockdown reduced ATM S1981 and CHK2 Thr68, indicating increased genome stability in A3B-deficient cells without Olaparib treatment. Olaparib treatment alone induced the phosphorylation of ATM, CHK2, p53, and BRCA1, which reflects the activation of dsDNA repair and checkpoint signaling pathways. Remarkably, the activation of the ATM/CHK2/p53 pathway induced by Olaparib is inhibited in the presence of A3B knockdown. Taken together, these data suggest that A3B knockdown diminishes both ATR-CHK1 and ATM-CHK2 pathways induced by Olaparib.

### A3B knockdown induces S/G_2_ arrest in PARPi-treated tumorspheres

It is well documented that the choice of DNA repair pathway depends on the cell cycle phase to allow sufficient time for DNA repair to occur^47^. Indeed, multiple studies have shown that promoting cell cycle progression can overcome PARPi resistance^48,49^. To evaluate the effects of A3B knockdown on the proliferating population, we performed flow cytometry of Ki67 and 7AAD in A2780 tumorspheres and adherent cells treated with or without Olaparib. In the absence of Olaparib, A3B loss induces a minor G_2_/M arrest in 2D cultured cells compared to the control condition, while tumorspheres are more prone to G_2_/M arrest as well as accumulation in S phase (Fig. S6), which is consistent with previous reports of preferred expression of A3B in G_2_/M phases^50^. In comparison, inhibition of A3B primarily leads to a less profound delay in S to G_2_ transition, but rather a strong depletion of S phase in both 2D cells and 3D tumorspheres in response to Olaparib (Fig. 5F,G), suggesting replication in S phase is uniquely impaired. We also noticed that shA3B-1 was more efficient at reducing S-phase cells compared to shA3B-2, likely due to more efficient A3B knockdown. These results imply that A3B alone induces mild replication stress in G_2_ phase, which is amplified in S phase when combined with PARPi.

### UNG modulates PARPi sensitivity in high A3B-expressing cells

**Fig. 6:**
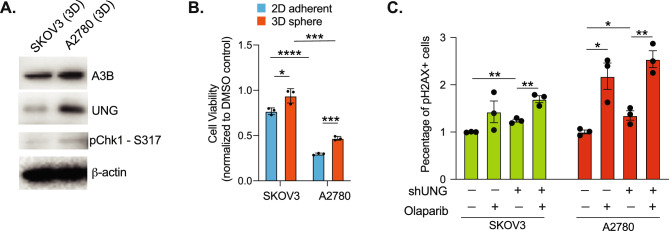
A3B induces PARPi sensitivity is not UNG-dependent. (**A**) Western blotting of A3B, UNG, and p-Chk1 (S317) in SKOV3 and A2780 tumorspheres. β-actin is used as loading control. (**B**) Cell viability of 2D adherent cells or 3D tumorspheres were quantified using CellTiter glo after Olaparib treatment (50 nM, 3 days). (**C**) Quantification of γH2AX in SKOV3 and A2780 3D tumorspheres after knockdown of UNG by shRNA (shUNG) and/or Olaparib treatment (50 nM, 3 days). Data was normalized to wildtype cells without Olaparib treatment. Error bars represent mean with SEM. *p < 0.05, **p < 0.01, ***p < 0.001, and ****p < 0.0001 unpaired Student t-test.

PARP1 quickly binds to ssDNA during DNA damage response, and consequently, PARP trapped on ssDNA by PARPi treatment can prevent completion of base excision repair (BER)^51,52^. Uracil generated by A3B C-to-U deaminase activity is readily converted to abasic sites (AP) by glycosylases such as uracil-DNA glycosylase (UNG), which are further processed in BER^53,54^. Depletion of UNG leads to synthetic lethality in cancer cells with high expression of A3B^55^. Thus, we reasoned that high A3B-expressing cells would rely on UNG-induced uracil excision to enhance the effects of PARPi. To test this hypothesis, we examined the response to Olaparib in two cell lines, SKOV3 and A2780, with varying A3B levels and sensitivity to A3B overexpression (Fig.S3A,F). High A3B expressing A2780 cells display an elevated level of phosphorylated Chk1 and UNG, suggesting these cells are under more intrinsic replication stress than low A3B expressing SKOV3 cells (Fig. 6A). Moreover, A2780 cells were more sensitive to Olaparib than SKOV3 in both 2D culture and 3D tumorspheres (~ 50% reduction in cell viability) (Fig. 6B). To directly assess the dependence of PARPi response on UNG activity, we performed shRNA-directed knockdown of UNG1/2 (shUNG) in both SKOV3 and A2780 and exposed these cells to Olaparib treatment (Fig. 6C). While knockdown of UNG alone results in only a slight increase in γH2AX level in both cell lines, Olaparib exacerbates this effect significantly in A2780, whereas SKOV3 is relatively unresponsive. However, we do not observe any significant difference in the induction of γH2AX by Olaparib between control and shUNG cells, suggesting the uracil repair by UNG may not be directly responsible for the enhanced PARPi sensitivity in high A3B expressing cells.

## Discussion

Using previously published scRNA-seq datasets, we analyzed the expression of A3 family genes in 6 HGSOC patients, including 1 with a *BRAC2* mutation. We reported HGSOC exhibits a high level of A3B, followed by A3C and A3A. We also reported that HGSOC tumorspheres maintain a relatively low level of A3B compared to adherent cells in PDX models and cell lines, while other A3s have similar expression regardless of CSC or non-CSC state. Similarly, a previous study using myeloproliferative neoplasm (MPN) as a model has demonstrated that mutational burden and A3-signature was lower in CD34 + MPN stem cells compared to those of MPN bulk peripheral blood^56^. Lastly, knockdown of A3B promotes tumorsphere formation capacity and leads to a significant increase in *SOX2* stemness factor, suggesting a delicate balance between A3B-induced tumor evolution and a stem-like state of self-preservation. It is worth noting that only one of the three PDX HGSOC models (OV033) readily forms tumorspheres, and it exhibits the highest level of A3B. We suspect additional molecular characteristics, such as the genetic alteration, BRCA mutation, and HRD status, likely contribute to tumorsphere formation capacity and A3B expression. Similarly, the majority of our studies were performed using the A2780 cell line. Future studies with PDX HGSOC with complementary genetic profiling, especially PARPi-resistant HGSOC lines, will be critical to identify if A3B activity is important for the response to PARPi.

An interesting aspect of CSCs is the heterogeneity and plasticity of this population, as non-CSCs can de-differentiate and gain CSC properties and vice versa. Since A3 expression and the associated mutational signatures are sporadic^19^, we are likely observing average gene expression patterns of A3B in a heterogeneous population of HGSOC. Future single-cell studies are needed to further determine whether the repression of A3B in CSCs is a stepwise transition or if A3B-high cells are eliminated during this process.

We next demonstrated that knockdown of A3B leads to Olaparib tolerance due to the lack of A3B-induced cellular replication stress in S cell cycle stages. Since PARPi triggers ssDNA lesions and replication stalling, we suspect that the accumulation of ssDNA will lead to new targeting regions for A3B and induce further DNA damage in the presence of DNA-damaging agents such as PARPi. Interestingly, we reported that loss of A3B relieves both ATR-CHK1 and ATM-CHK2 signaling, suggesting A3B is associated with both replication stress and genome instability in the presence of PARPi. However, the G2/M phase accumulation persists in shA3B cells. Thus, other compensatory DNA repair mechanisms, such as p38-MAPK, could sustain the progress through later phases of the cell cycle. Future studies are necessary to determine the contribution of these pathways in relevant model systems.

Recent studies have shown that A3B overexpression is associated with improved clinical outcomes of platinum therapy^57^. A3B expression and ongoing DNA mutational activity may provide important biomarkers in predicting PARPi response in HGSOC and other malignancies. This should be achieved by analyzing large cohorts of PARPi-resistant and -responsive patients while also considering the genomic status of *BRCA1/2* mutations and other DNA repair mechanisms. Our findings also suggest that future studies should explore how to balance the beneficial effects of overexpressing A3B with the harmful effects of DNA mutagenesis. The development and application of A3B inhibitors should be carefully considered and timed, especially when used in combination with DNA-damaging agents such as PARPi.

## Experimental methods

### Cell culture

A2780 (Sigma, Cat # 93,112,519; RRID:CVCL_0134) and SKOV3 (ATCC, Cat #HTB-77; RRID:CVCL_0532) human cell lines were cultured in 37 °C in RPMI 1640 supplemented with 10% FBS, 2 mM L-glutamine and 1X penicillin–streptomycin. All cell lines were confirmed to be mycoplasma-free with repeated testing and authenticated by short-tandem repeat (STR) profiling. A3B knockdown A2780 cell line was generated by transducing A2780 cells with lentivirus containing either a scramble control shRNA or shRNA targeting A3B labeled with EGFP. Cells were sorted based on EGFP marker and cultured as wildtype A2780 cells. Stable A3B knockout was confirmed by western blot every 5 passages.

### Antibodies

The following antibodies were used: APOBEC3B (Abcam, Cat #ab184990; RRID:AB_2891094); CD133-APC (Biolegend, Cat #397,906; RRID:AB_2876721); SOX2 (Abcam, Cat #ab92494; RRID:AB_10585428); PARP (Abcam Cat #ab191217; RRID:AB_2861274); Cleaved PARP (Abcam Cat #ab32561; RRID:AB_777103); γH2AX (S139) (Abcam, Cat #ab11174; RRID:AB_297813); Chk1 (Abcam, Cat #ab40866; RRID:AB_726820); pChk1 (S317) (Abcam, Cat #ab278717); UNG (Origene, Cat #TA503563; RRID:AB_11126624); pATR (S428) (Cell Signaling, Cat #2853); pATM (S1981) (Cell Signaling, Cat #13,050); pChk2 (Thr68) (Cell Signaling, Cat #2197); phosphor-p53 (S15) (Cell Signaling, Cat #9286); pBRCA1 (S1524) (Cell Signaling, Cat #9009); beta-actin (Abcam, Cat #ab8227; RRID: AB_2305186); Goat anti-Rabbit Alexa488 secondary antibody (ThermoFisher, Cat #A32731; RRID: AB_2633280); Goat anti-Rabbit Alexa647 secondary antibody (ThermoFisher, Cat #32,733; RRID: AB_2633282).

### Lentiviral shRNA construct, transduction, and transfection

Lentiviral vectors scramble control (pLV-shRNA-EGFP, VB151023-10,034), shRNA targeting A3B (shA3B-1: pLV [shRNA]-EGFP:T2A:Puro-U6 > hAPOBEC3B[shRNA#1], VB200311-6578csf; shA3B-2: pLV[shRNA]-EGFP:T2A:Puro-U6 > hAPOBEC3B[shRNA#2], VB200312-7989fkm), and shRNA targeting UNG (shUNG: pLV[shRNA]-EGFP:T2A:Puro-U6 > {shUNG1/2}, VB210323-1052pzu) were purchased from VectorBuilder. The titers were determined by transduction of HEK293T cells and assessment of EGFP by flow or RT-qPCR of the 5’ LTR region. Lentiviral transduction of cell lines was performed at a MOI of 10–25. The cells were seeded in 96-well plate (2.5 × 10^5^ cells per well) in 100 μL of media for 24 h before adding lentivirus. After incubation with lentivirus for 2–3 days, EGFP^+^ cells were sorted to isolate successfully transduced cells.

A3B overexpression plasmid was transfected using Lipofectamine 3000 (Thermo Fischer Scientific) following the manufacturer’s instruction. Briefly, A2780 tumorspheres were seeded in 96-well plate (2 × 10^5^ cells per well) in 100 μL of media. A3B overexpression plasmid (20 ng) was mixed with lipofectamine 3000 reagents in Opti-MEM medium and incubated at room temperature for 20 min, and then added to the cell culture for 24 h.

### Animal experiments

All mouse studies were conducted under protocols approved by the Institutional Animal Care and Use Committee (IACUC) of the University of California, San Diego and were in compliance with federal regulations regarding the care and use of laboratory animals: Public Law 99–158, the Health Research Extension Act, and Public Law 99–198, the Animal Welfare Act which is regulated by USDA, APHIS, CFR, Title 9, Parts 1, 2, and 3. In addition, the principle of ARRIVE guidelines were considered and all methods are in accordance with ARRIVE guidelines to ensure the high standard of reproducibility. Mice engrafted with HGSOC PDX models were purchased from The Jackson Laboratory (Table S1) and maintained in the Sanford Consortium for Regenerative Medicine vivarium according to IACUC approved protocols. Mice were humanely sacrificed by CO_2_ inhalation in an airtight euthanasia chamber followed by cervical dislocation. The CO_2_ was filled at a rate of 30–70% of the chamber volume per minute. Mice were between 8–12 weeks of age and weighted approximately 20–30 g when they were sacrificed. Tumors were collected for downstream analysis.

For Olaparib treatment, A2780 cells successfully transduced lentiviral scramble control or shRNA targeting A3B (shA3B) were cultured as tumorspheres for three days. Cells were collected and washed twice with ice-cold PBS. 6–8 weeks Rag2^-/-^γc^-/-^ old female mice were injected with 1 × 10^6^ cells suspended in 100 μl sterile PBS subcutaneously in the hind leg. 10 days after the injected, mice were randomized into the two treatment groups: vehicle (PBS) and Olaparib (50 mg/kg daily) via the IP route for 14 days. Tumor sizes were measured 2–4 times per week using a caliber to track tumor sizes and tumor weights were measured at the end of experiments.

### Tumorsphere formation assay

PDX tumors were minced, and supernatant was collected by washing the tissues 23 times with cold PBS buffer. If large pieces are still visible, tissues are digested with 1 mg/mL collagenase and incubated on an orbital shaker for 2–3 h. The remaining tissues were filtered out by passing through a 100 m filter. To form 3D tumorspheres, cells were seeded at 1 × 10^5^ cells/mL in ultra-low attachment 6-, 24-, or 96-well plates (Costar) in mammocult complete media (StemCell Technologies) and incubated at 37 C and 5% CO_2_. After 7 days, spheres were counted and plated in ultra-low attachment plates for downstream assays.

### Olaparib treatment and MTT assay

2D adherent cells or 3D tumorspheres were plated in 12-, 24-, or 96-well plates at 1 × 10^5^ cells/mL density. Increased concentrations of Olaparib (Selleck, Cat #NC0968989) were added in growth media. After 5 days, cell viability was determined using MTT assay (Sigma Millipore, Cat #11,465,007,001) according to manufacturer’s protocol. In short, 100 μL of cell culture was transferred to a 96-well plate and 100 μL of MTT labeling reagents were added. After incubation at 37^0^C for 4 h, 100 μL of solubilization solution was added to incubate at 37^0^C overnight. Viability was analyzed by quantifying the absorbance using a microplate reader.

### ALDH activity assay

ALDH activity was measured using ALDEFluor fluorescent assay (Stemcell Technologies, Cat #01,700) or ALDERed assay kit (Millipore, Cat #SCR150) for EGFP + cells. 2D adherent cells and tumorspheres were collected by centrifugation (300 g, 5 min) and dissociated into single cell suspension by incubation with 0.01% trypsin for 5 min at room temperature. Cells were resuspended in ALDEFluor assay buffer containing ALDH substrate and incubated with or with ALDH inhibitor DEAB for 45 min in 37^0^C. After incubation, samples are washed twice with ice-cold ALDHFluor assay buffer and analyzed on a flow cytometer (BD FACSAria II or Fortessa X-20). If combined with CD133 cell surface staining, cells were blocked using FcR block (Biolegend, San Diego, CA) for 15 min before adding CD133 antibody staining to a final dilution of 1:25 and incubated for 30 min on ice in the dark.

### γH2AX flow cytometry

Cells cultured in 2D or 3D conditions were harvested by trypsinization for 5 min at room temperature and resuspended in single cell suspension and kept on ice for remaining of the staining procedure. Cells were stained with ethidium monoazide (EMA) for 15 min in the dark and then 15 min under light. After washing in staining buffer, cells were fixed and permeabilizated with an intracellular buffer set (eBioscience, San Diego, CA) and intracellularly stained with an antibody against γH2AX-Alexa 647 at 1:50 dilution for 30 min in the dark. After staining, cells were washed twice with PBS with 2% FBS, and resuspended in 100–300 μL of the same buffer for analysis on flow cytometer.

### Cell cycle flow cytometry

2D or 3D cells were collected into single cell suspension as described above, fixed in 70% ethanol and stored in—20^0^C overnight. Cells were then washed twice and resuspend into ice-cold PBS with 2% FBS and 50 g/mL DNAse-free RNAse (Qiagen). Ki67 antibody was added at 1:50 dilution and incubated with cells for 45 min on ice in the dark. Cells were washed twice with PBS with 2% FBS and resuspended in 100–300 μL of buffer containing 5 g/mL of 7-AAD. Flow analyses were performed as described above.

### Immunofluorescent imaging of γH2AX

For 2D adherent cells, IF staining was performed in the culture plates. For tumorspheres, all staining steps were performed in 1.5 mL Eppendorf tubes by centrifugation and aspirating using a pipet. Cells were fixed with 100% methanol for 10 min at room temperature, permeabilized with 0.1% Triton X100 for 10 min at room temperature, and then blocked with 5% BSA in 0.1%PBS-Tween for 2–3 h on a rocker. The cells were then incubated with primary antibody of γH2AX (0.1 µg/mL) at 4 °C overnight. After washing three times with PBS-0.1% Triton X100 buffer, secondary antibody to Rabbit IgG (1:1000 dilution) was added and incubated for 2 h at room temperature while rocking. Images were acquired on Nikon Confocal microscope.

### RNA extraction and quantitative real-time polymerase chain reaction

Total RNA was isolated using RNeasy Micro kit or Mini kit (Qiagen) and the quality was determined by NanoDrop. Complementary DNA was synthesized according to published methods^58^. qRT-PCR was performed in duplicate or triplicate on an CFX384 with the use of SYBR GreenER qPCR SuperMix (Invitrogen), 5 ng of template cDNA and 0.2 µM of each forward and reverse primer**.** Human specific HPRT primers were used as housekeeping control. Quantitative values were obtained from the cycle number (Cq value) using the Bio-Rad Maestro Software. The RT-qPCR primers are shown in Table S3.

### Western blots

Cell lysate (10 ug) was mobilized onto a nitrocellulose membrane after electrophoresis on a 10% SDS- acrylamide gel. The membrane was blocked in 5% BSA/20 mM Tris–HCl for 30 min. The blot was incubated with primary antibody in 5% BSA/20 mM Tris–HCl/0.1% Tween20 overnight at 4 °C, followed by secondary HPR-linked Rabbit or mouse IgG antibody (Cell Signaling) for 2 h at room temperature. Membrane was then incubated in SuperSignal West Femto Substrate (ThermoFisher) for chemiluminescent reading on ChemiDoc System (Bio-Rad).

### Whole RNA-sequencing

Samples with RNA integrity numbers (RIN) ≥ 7 will be processed using SMART cDNA synthesis and NEBNext paired-end DNA Sample Prep Kit to prepare libraries. RNA-sequencing were performed on NovaSeq 6000 S4 with 150 bp paired-end reads.

### Transcript and gene quantification and differential expression

Reads were trimmed for adapters with fastp^59^ and aligned to the GRCh38.96 reference transcriptome with kallisto^60^. Gene-level count summaries were created from transcript-level counts using a custom R script based on mygene^61^. Differential expression analyses were carried out using limma^62^.

PCA plots and heatmaps were created in Qlucore Omics Explorer (version 3.9). Multi-group ANOVA was used to identify top 500 differentially expressed genes, ordered by q-value. Gene set enrichment analysis was performed using the 304 uniquely differentially expressed genes in shA3B-1 treated with Olaparib compared to DMSO, using both using R (version 4.3.1), with packages ClusterProfiler (version 4.10.1) and Enrichplot (version 1.22.0) as well as the GSEA application provided by Broad Institute and UC San Diego^63^. For enrichment plots made in R, log_2_(fold change) was provided as input. For gene sent enrichment analysis performed in the GSEA application, the KEGG Legacy (version 2024.1.Hs) data set from MSigDb was utilized^64–66^.

### Other statistical analysis and reproducibility

All experiments were performed with at least two biological or experimental replicates, with specific number of replicates stated in the figure legends. Unless otherwise stated, the statistical analyses were performed using GraphPad Prism (v7.0) and statistical significance were determined at p value < 0.05, with specific statistical test stated in the figure legends.

## Supplementary Information

Supplementary Information 1.

Supplementary Information 2.

Supplementary Information 3.

## Data Availability

The RNA-sequencing dataset used in this study is available at GEO database (GEO309870).
