# Supplementary figures and images for: APOBEC3B enhances the efficacy of PARP inhibitors in elimination of ovarian cancer stem cell
Source: Sci Rep. 2026 Jan 14;16:5194. doi: 10.1038/s41598-026-35939-y (PMC12881425; doi:10.1038/s41598-026-35939-y)

Fig. 2A

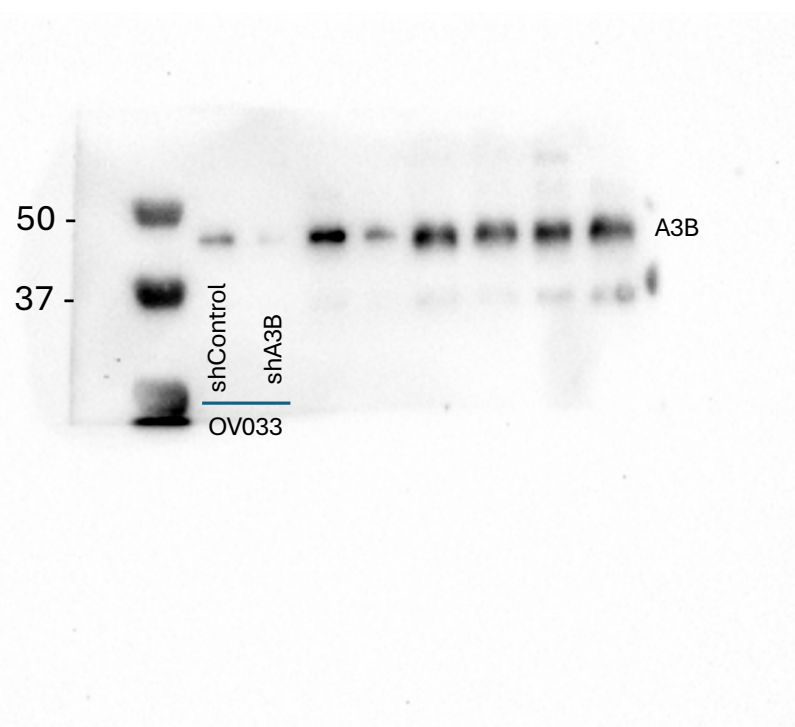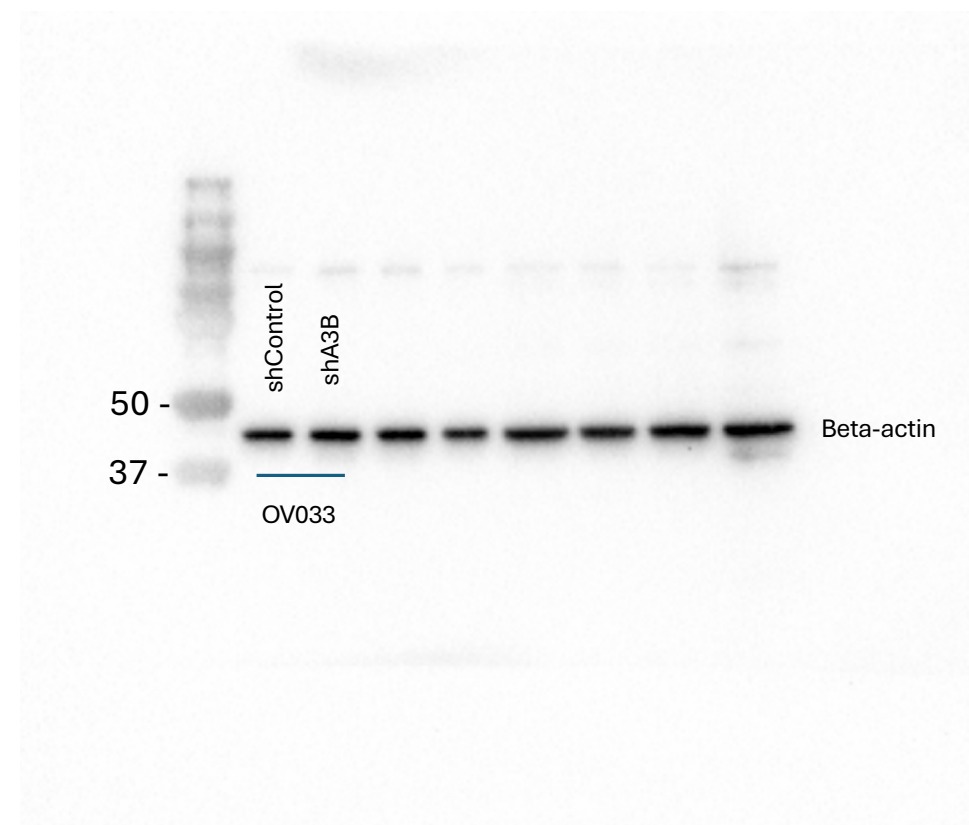

Fig. 5D

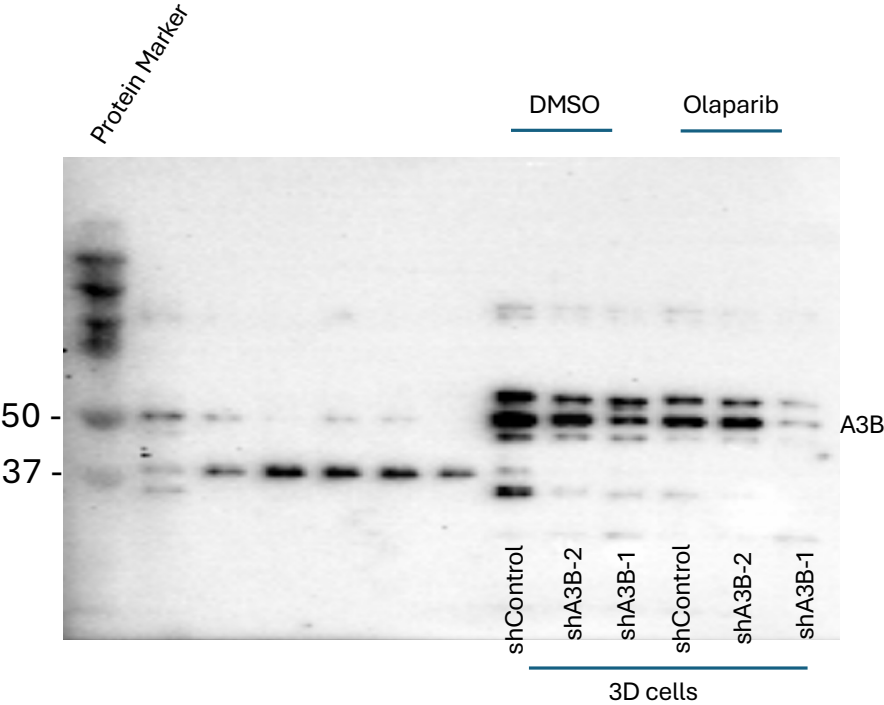

Fig. 5D

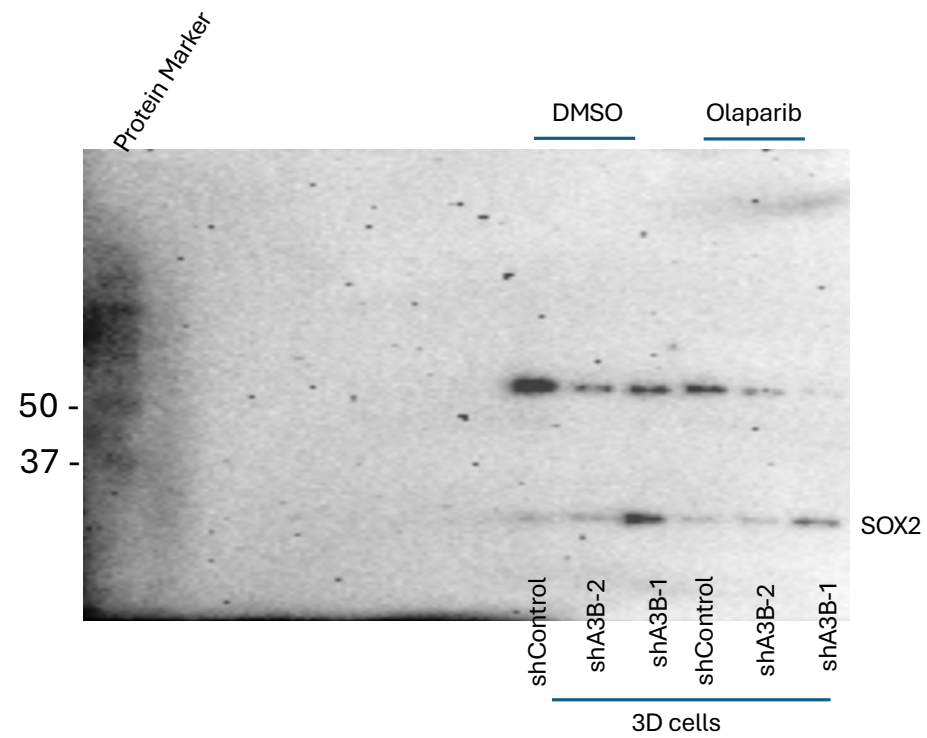

Fig. 5D

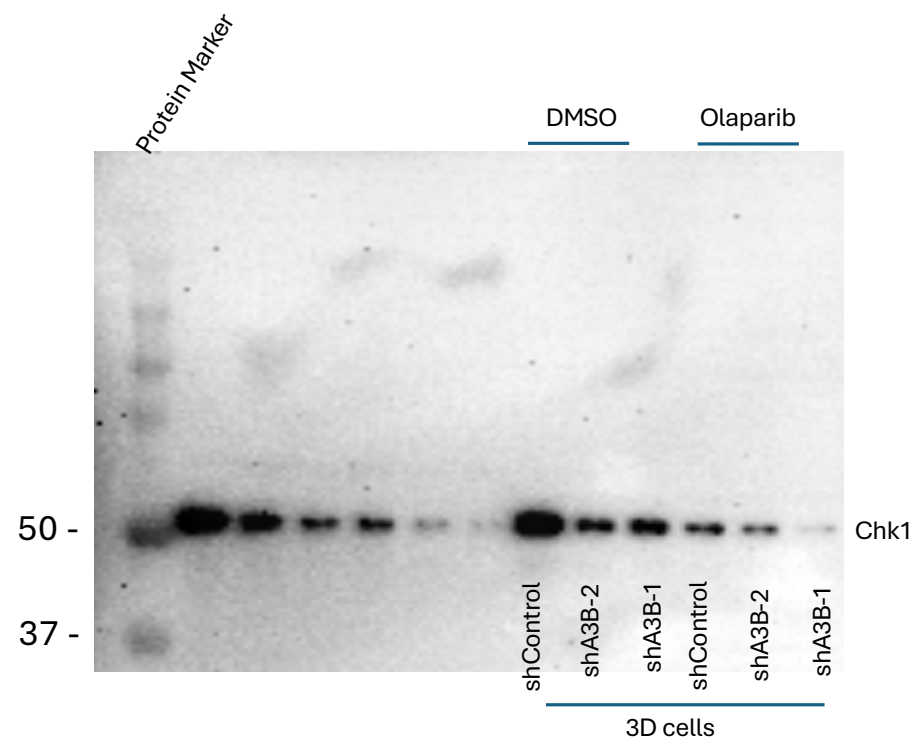

Fig. 5D

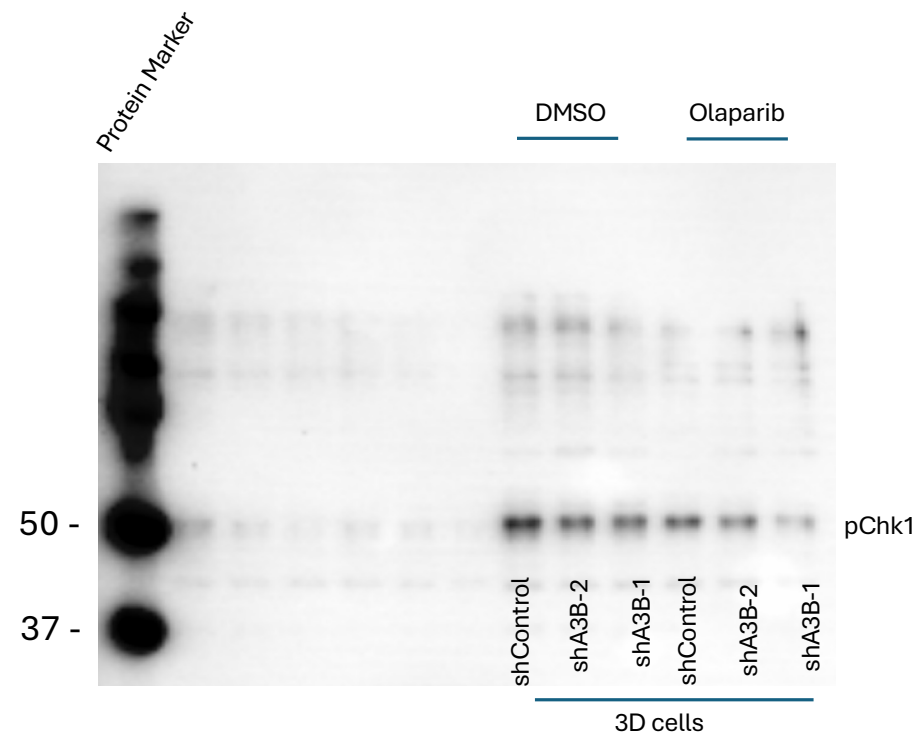

Fig. 5D

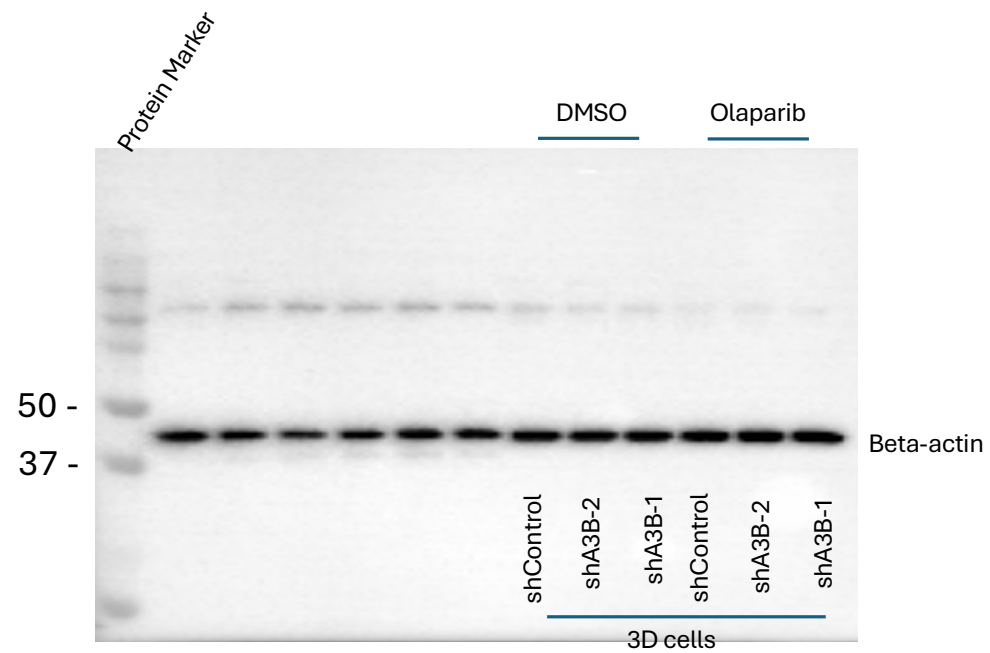

Fig. 5E

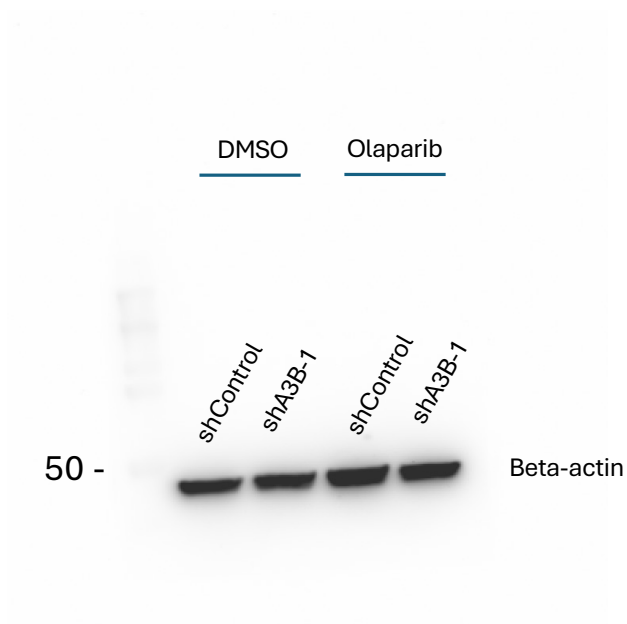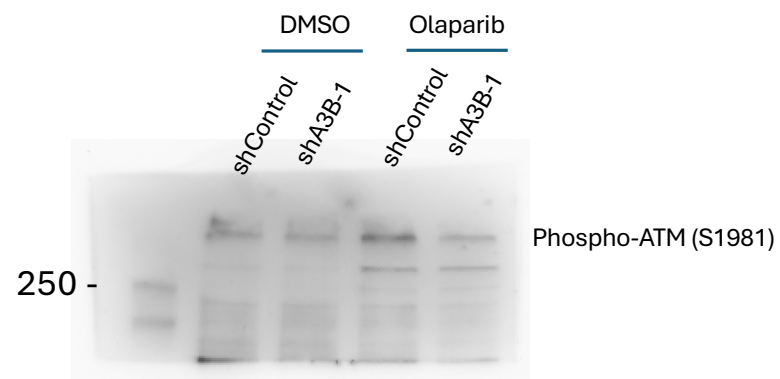

Fig. 5E

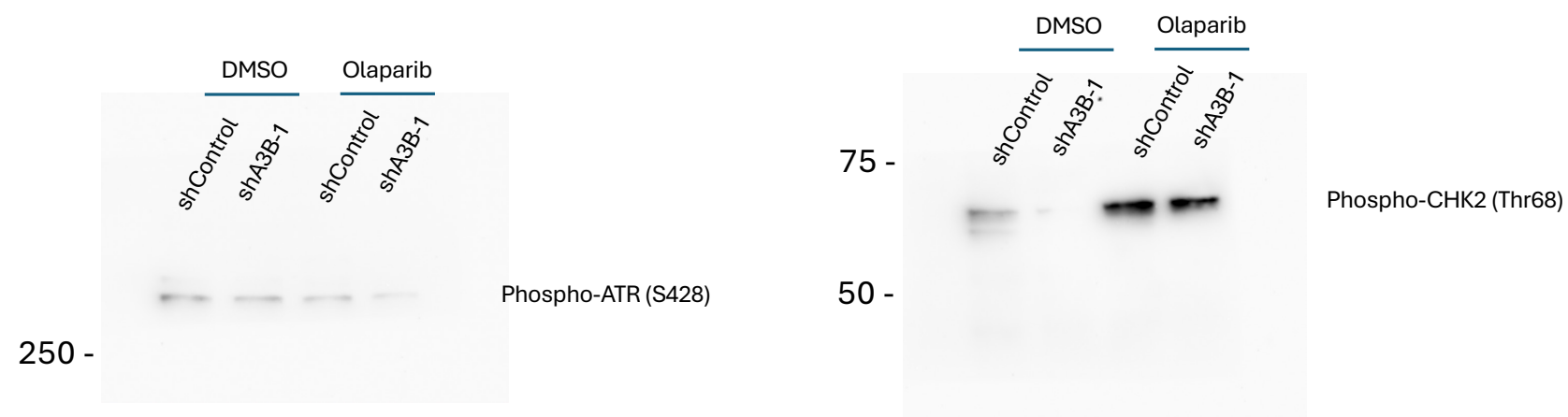

Fig. 5E

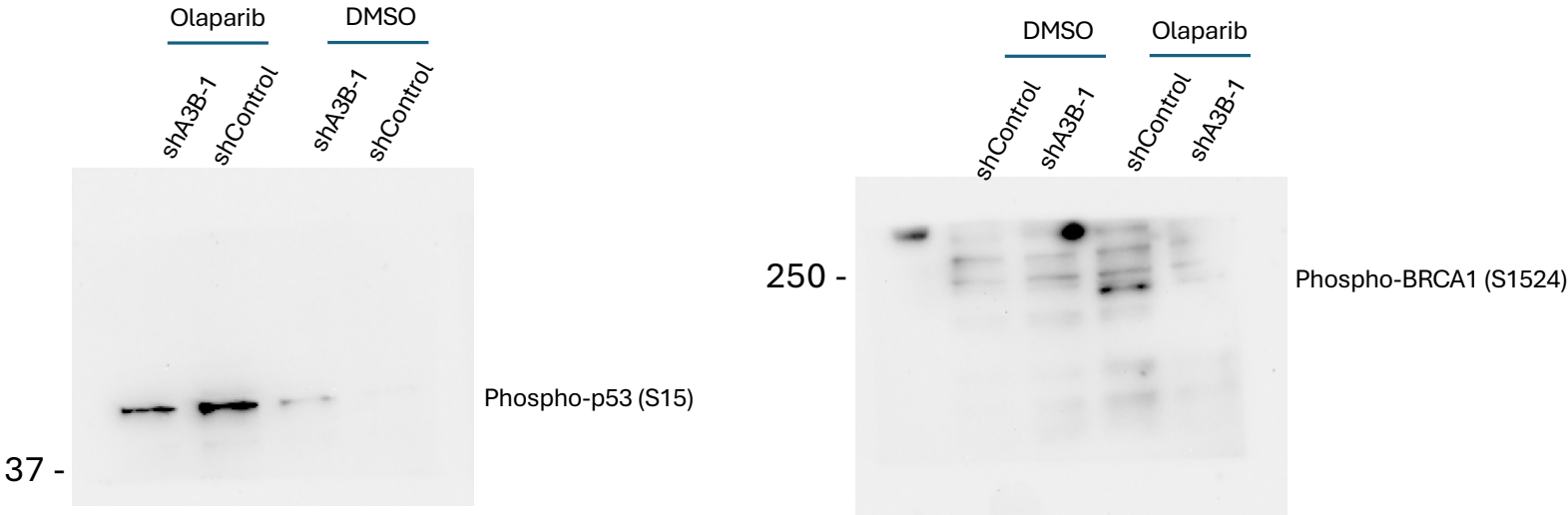

Fig. 6A

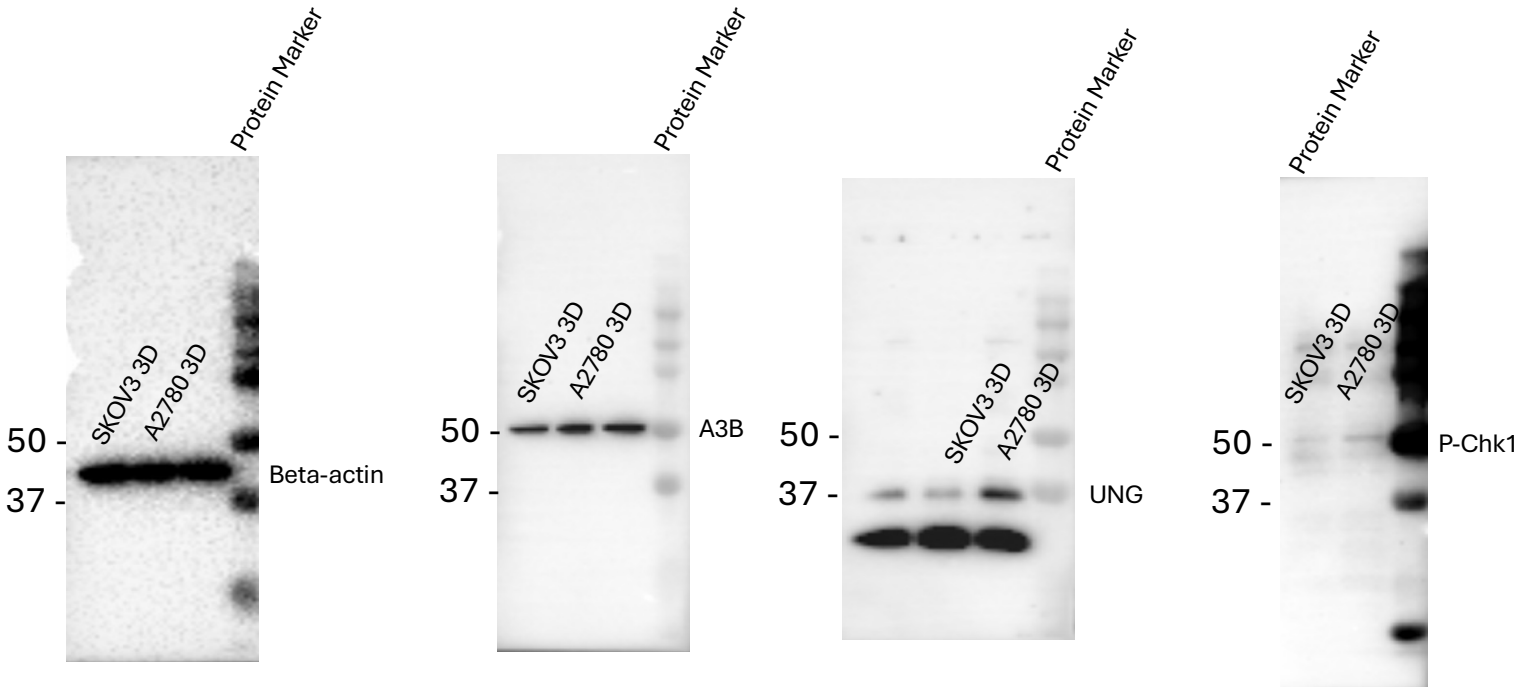

Fig. S2D

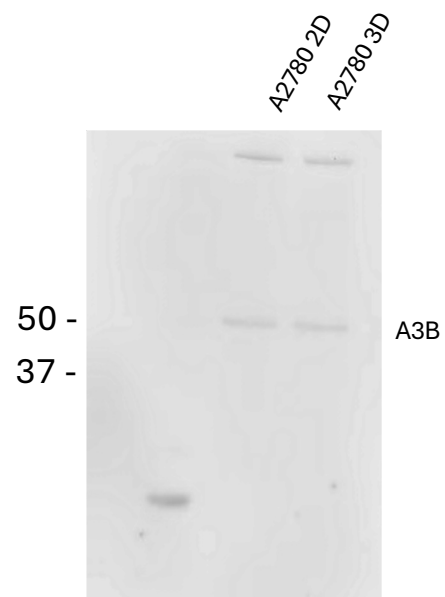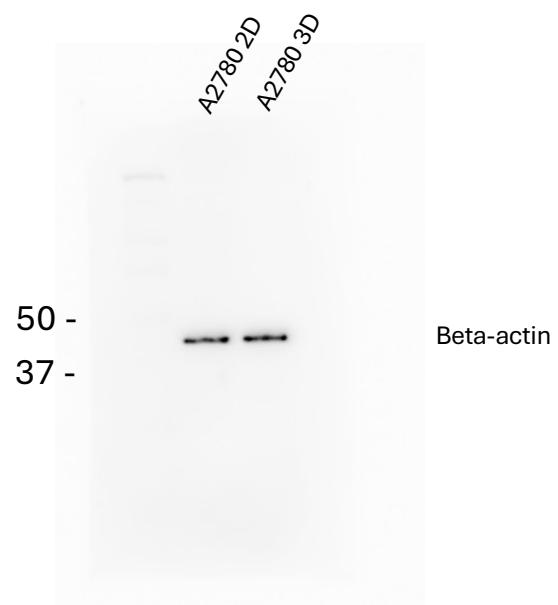

Fig. S5C

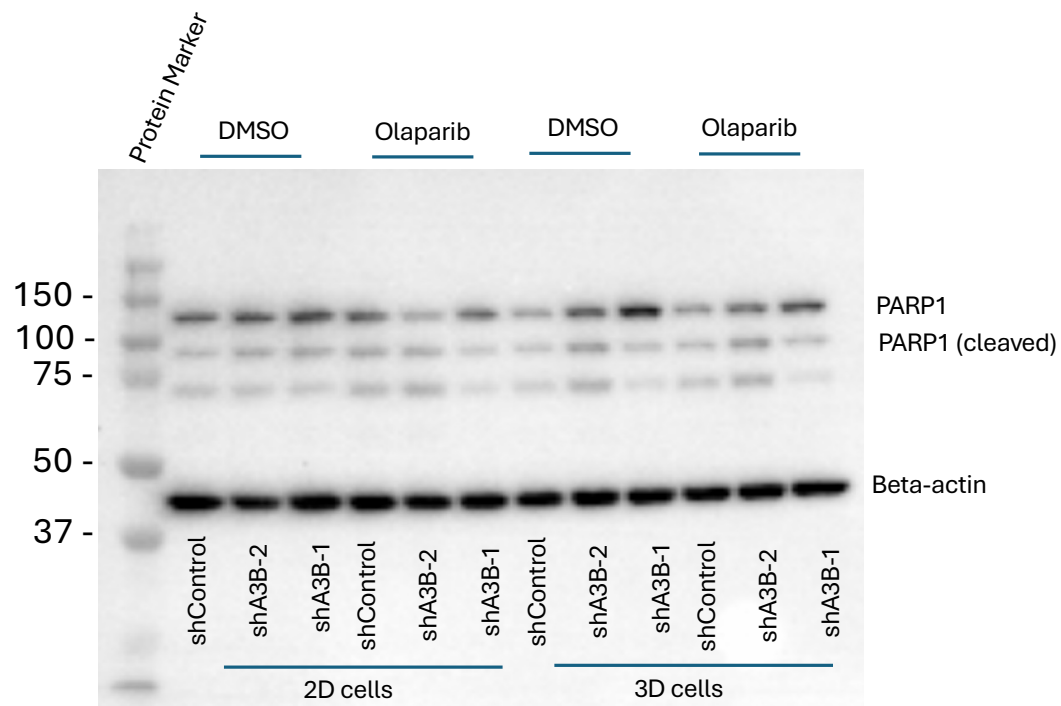

Fig. S5D

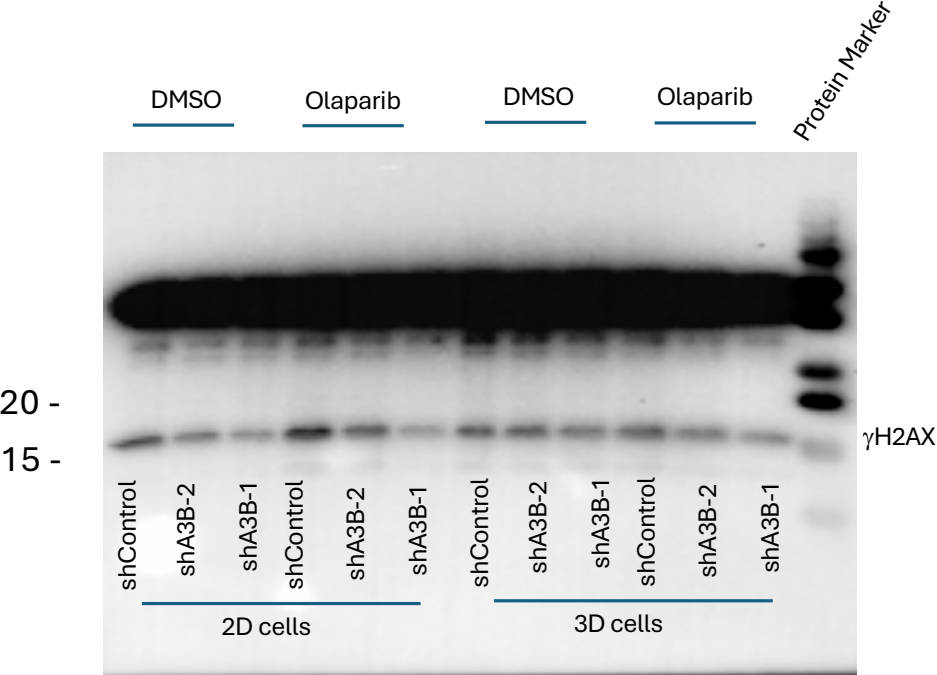

Fig. S5D

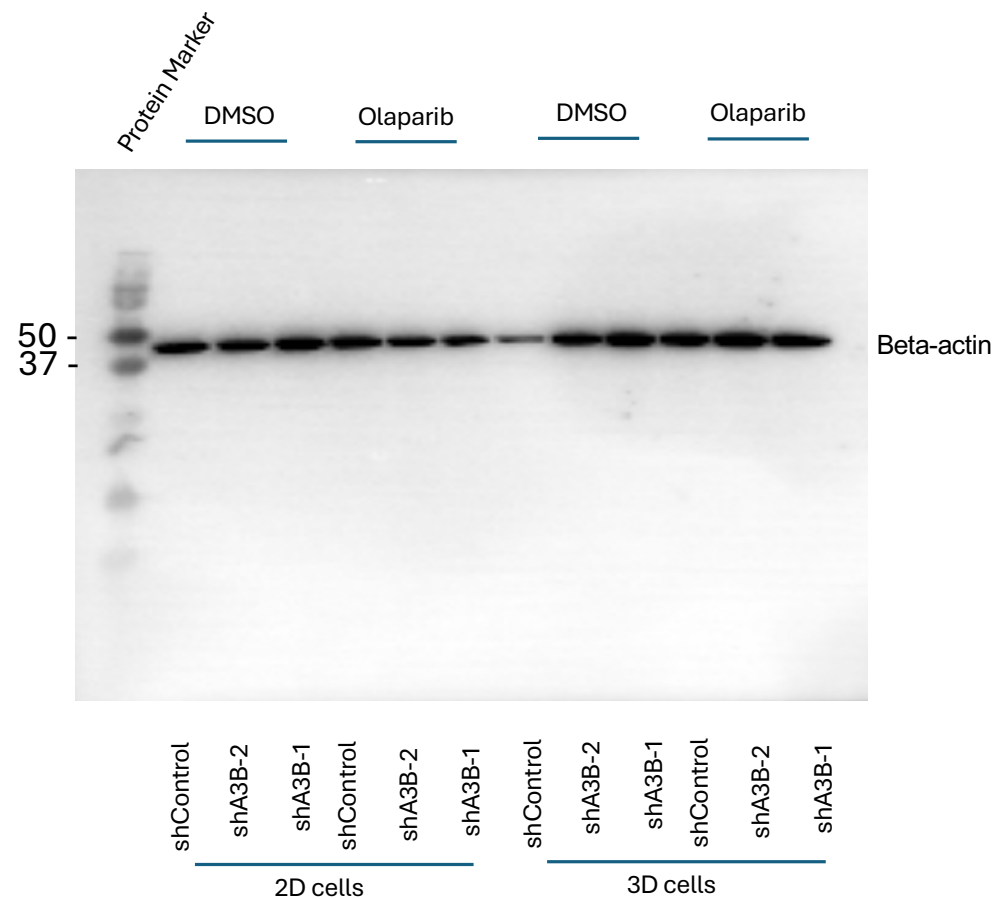

Supplement: Supplementary file 1 — Supplementary Information 1. [file 41598_2026_35939_MOESM1_ESM.pdf]
