# Supplementary material for: APOBEC3B enhances the efficacy of PARP inhibitors in elimination of ovarian cancer stem cell
Source: Sci Rep. 2026 Jan 14;16:5194. doi: 10.1038/s41598-026-35939-y (PMC12881425; doi:10.1038/s41598-026-35939-y)
Supplement: Supplementary file 2 — Supplementary Information 2. [file 41598_2026_35939_MOESM2_ESM.pdf]

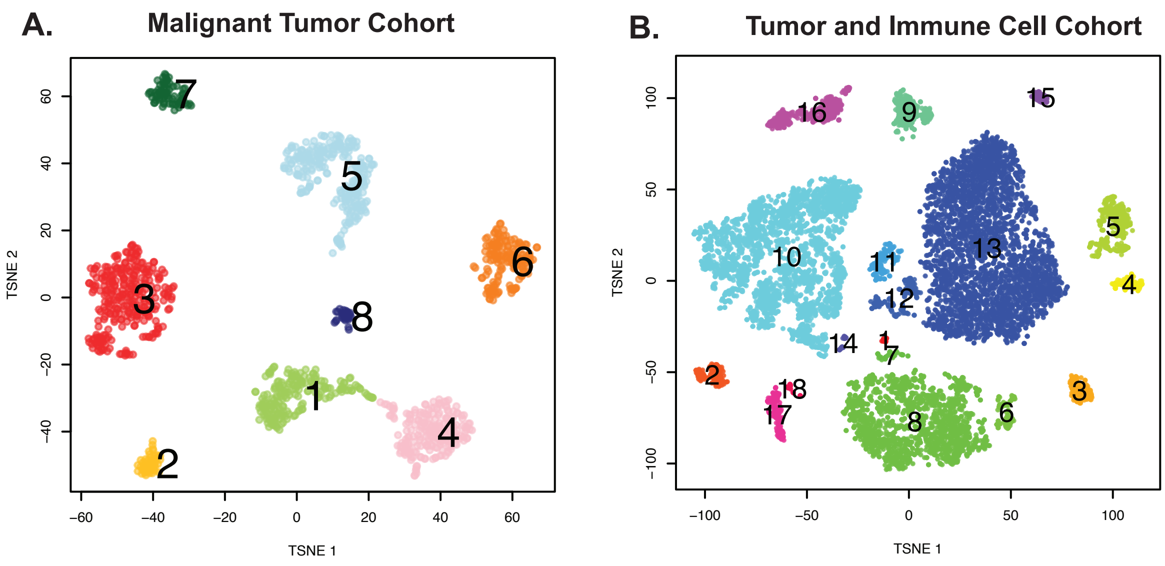

Cell Type	Cluster #
M2	10
M1	10, 11
Ery	18
TC	17
BC	16
DC	14, 15
M	10, 11, 12, 13
CAF	6, 7, 8, 9
OC	1, 2, 3, 4, 5

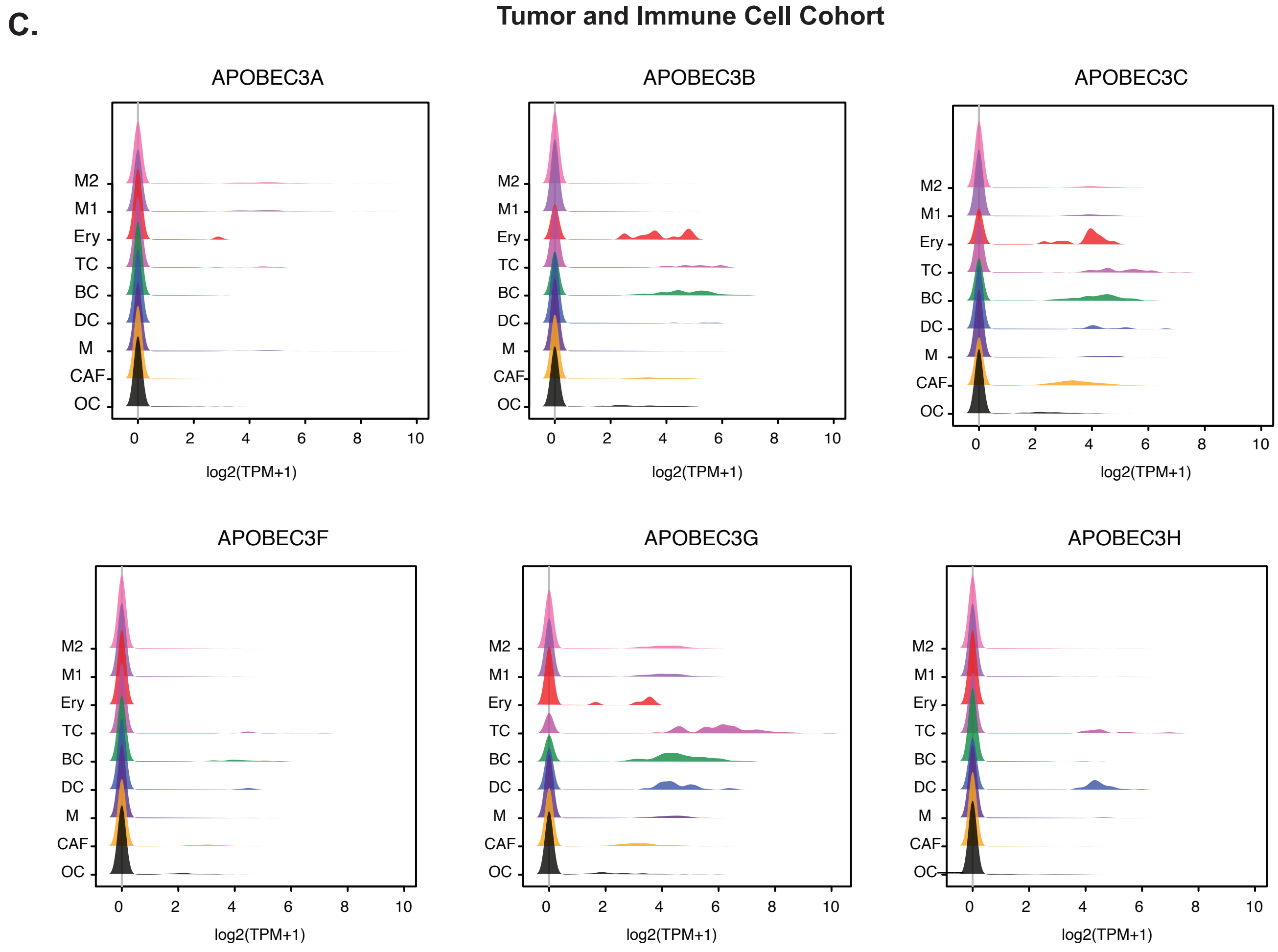

Figure S1, Expression of APOBEC3 family members in immune cells of HGSOC, related to Figure 1. A. t-SNE plot of EPCAM+CD24+ enriched tumor cells from 8 HGSOC ascite patients. Patient 7 is BRAC2 muated.. **B.** t-SNE plot of 6 HGSOC ascite samples; compose of both HGSOC tumor and immune cells. **C.** Ridgeplots showing expression of APO-BEC3s in various immune cells discovered in HGSOC ascite samples, in the cohort shown in **B.** (n = 6 samples) M2: M2 macrophages; M1: M1 macrophages; Ery: erythrocytes; TC:T cells; BC:B cells; DC: dendritic cells; M: macrophages; CAF: fibroblasts; OC: Ovarian Cancer Cells.

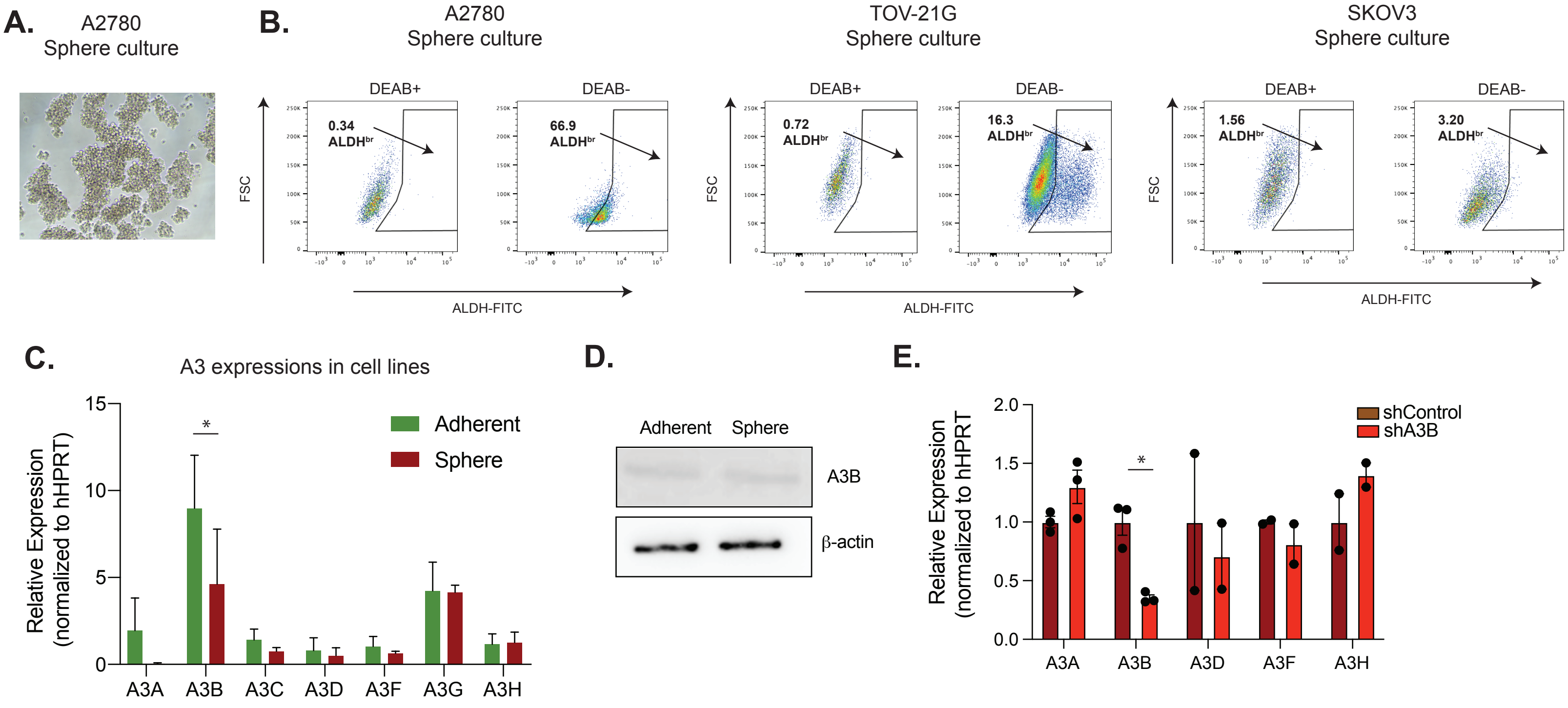

Figure S2, APOBEC3B is upregulated in HGSOC cell lines, related to Figure 2. A. Representitive image of 3D tumorsphere of A2780 cell line. 20x magnification. **B**. Levels of ALDH^{br} CSC in tumorsphere of A2780, TOV-21G and SKOV3 measured by flow cytometry. **C**. Differential expressions of A3s were compared between 2D adherent culture and 3D tumorsphere culture in SKOV3, TOV-21G, and A2780. *p<0.05, unpaired student t-test. **D**. Western blot showing A3B expression in adherent and sphere culture of A2780. **E.** Expression of A3 family genes after A3B knockdown in A2780 3D tumorspheres (n = 2-3 independent experiments). *p<0.05, unpaired student t-test.

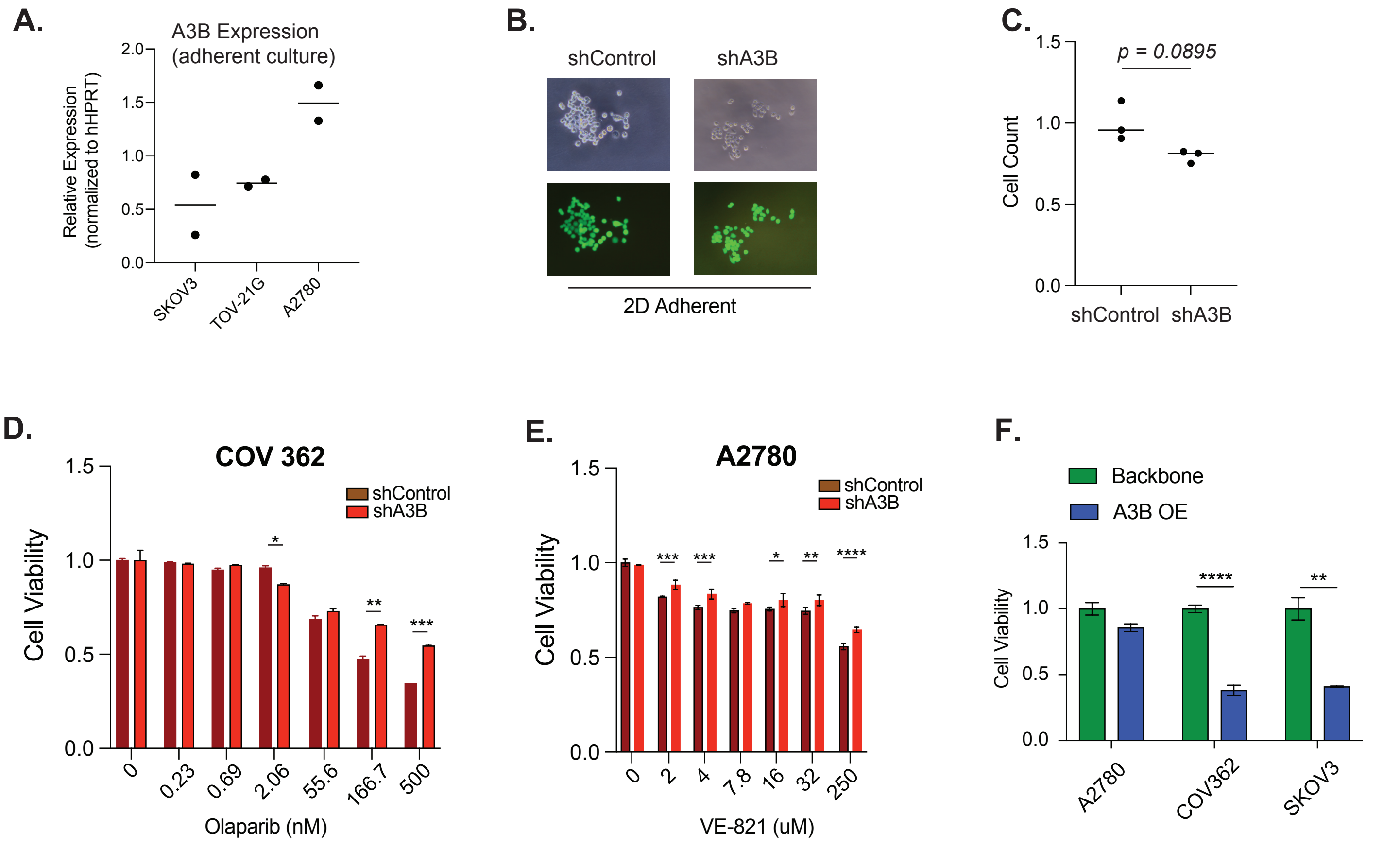

Figure S3, Knockdown of A3B increases sensitivity to PARPi Olaparib, related to Figure 3. A. Expression of A3B was quantified by RT-qPCR in three HGSOC cell lines. n = 2 experiments. **B**. Representitive image of 2D culture of A2780 cell line. 20x magnification. **C**.A2780 cell numbers were determined after A3B knockdown after 5 days. **D.** Cell viability was quantified in shControl and shA3B COV362 cells treated with Olaparib at various concentrations (n = 2). **E.** Cell viability was quantified by MTT assay in A2780 3D spheres treated with ATRi VE-821 (n = 3). **F.** Cell viablity in A3B overexpressing cells. A2780 was transfected wit 20 ng of plasmid, and COV362 and SKOV3 were transfected with 5 ng of plasmid (n = 3). *p<0.05, **p<0.01, ***p<0.001, ****p<0.0001, unpaired Student t-test

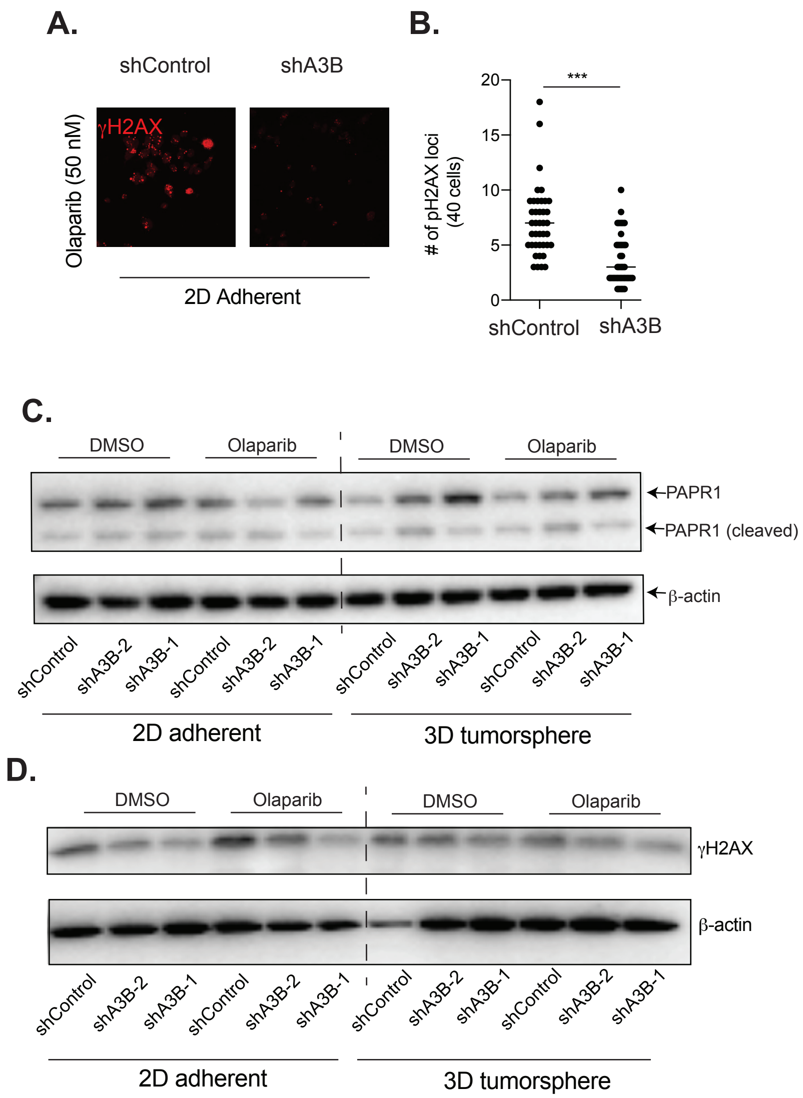

Figure S5, Knockdown of A3B increases sensitivity to PARPi Olaparib, related to Figure 5. A. Representative immunofluorescent imaging of γH2AX in shControl and shA3B 2D adherent cells after treatment with Olaparib (50 nM) in A2780. **B**. Quantification the number of γH2AX+ loci in 40 cells (D). **C**. Western blot showing PARP1 and cleaved PARP1 level in A2780 cells treated with DMSO or Olaparib (50 nM). **D**. Western blot γH2AX in 2D and 3D A2780 cells treated DMSO or Olaparib (50nM). ***p<0.001, ****p<0.0001, unpaired Student t-test

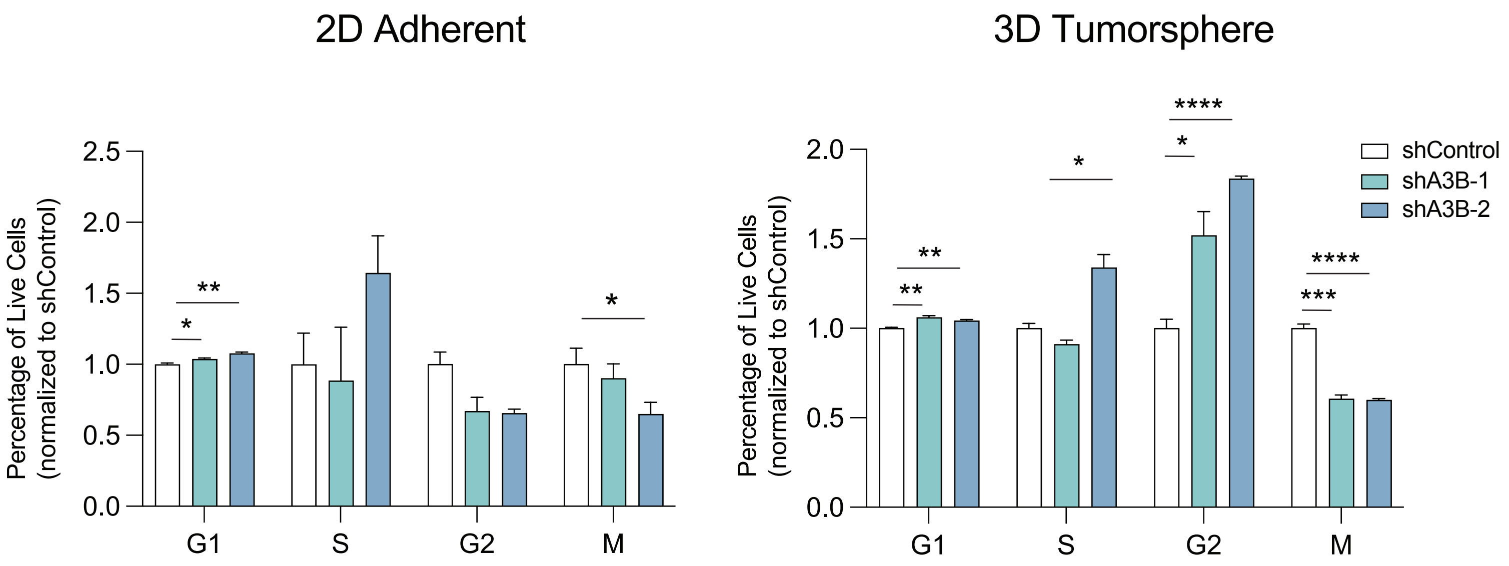

Figure S6. A3B loss induces S/G2 cell cycle arrest in HGSOC tumorspheres, related to Figure 5. A2780 cells transduced with control shRNA lentivirus or lentivirus targeting A3B were cultured in 2D adherent or 3D tumorsphere conditions. Cell cycle status were determined based on Ki67 and 7AAD flow analysis. n = 3 experimental triplicates. Unpaired student t-test.
