# Supplementary material for: APOBEC3B enhances the efficacy of PARP inhibitors in elimination of ovarian cancer stem cell
Source: Sci Rep. 2026 Jan 14;16:5194. doi: 10.1038/s41598-026-35939-y (PMC12881425; doi:10.1038/s41598-026-35939-y)
Supplement: Supplementary file 3 — Supplementary Information 3. [file 41598_2026_35939_MOESM3_ESM.pdf]

SUPPLEMENTAL INFORMATION

APOBEC3B enhances the efficacy of PARP inhibitors in elimination of ovarian cancer stem cell

Maria Rivera, Lucy Liu, Sabina Enlund, Chae-Eun Lim, Haoran Zhang, Kaifu Yang, Roman Sasik, Leslie A. Crews, Kathleen M Fisch, Ramez N. Eskander, Frida Holm, Qingfei Jiang*

* Corresponding Authors:
Qingfei Jiang, PhD, q1jiang@health.ucsd.edu

SUPPLEMENTAL TABLES

Table S1: Patient characteristics of PDX models

Table S2. Differentially expressed gene lists of A3B knockdown and Olaparib treated A2780 tumorspheres.

Table S3. Primers for RT-qPCR used in this study, related to STAR Methods.
